# Evidence for a functional interaction between yeast Pol ε and PCNA *in vivo*

**DOI:** 10.1093/nar/gkaf1339

**Published:** 2025-12-17

**Authors:** Noopur Singh, Roni Odai, Ulf Persson, Göran O Bylund, Ikenna Obi, Nasim Sabouri, Gemma C Atkinson, Erik Johansson

**Affiliations:** Department of Medical Biochemistry and Biophysics, Umeå University, 901 87 Umeå, Sweden; Department of Experimental Medical Science, Lund University, 223 62 Lund, Sweden; Department of Medical Biochemistry and Biophysics, Umeå University, 901 87 Umeå, Sweden; Department of Medical Biochemistry and Biophysics, Umeå University, 901 87 Umeå, Sweden; Department of Medical Biochemistry and Biophysics, Umeå University, 901 87 Umeå, Sweden; Department of Medical Biochemistry and Biophysics, Umeå University, 901 87 Umeå, Sweden; Science for Life Laboratory, Umeå University, 901 87 Umeå, Sweden; Department of Experimental Medical Science, Lund University, 223 62 Lund, Sweden; Science for Life Laboratory, Lund University, 223 62 Lund, Sweden; Department of Medical Biochemistry and Biophysics, Umeå University, 901 87 Umeå, Sweden

## Abstract

DNA replication relies on precise coordination between proteins, including the sliding clamp proliferating cell nuclear antigen (PCNA), which encircles DNA to interact with key players in replication and repair. While biochemical studies have demonstrated interactions between PCNA and DNA polymerases δ and ε during DNA synthesis, the functional role of the Pol ε–PCNA interaction *in vivo*, particularly during leading strand synthesis, remains to be elucidated. To address this question, we employed AlphaFold to model how PCNA interact with four-subunit yeast Pol ε. Our models revealed two distinct points of interaction between Pol ε and PCNA: one at the P-domain and another at a PIP-box, a classical PCNA interaction motif. To validate these findings, we generated mutants that disrupted the Pol ε–PCNA interaction interface. Biochemical assays demonstrated that the PIP-box is critical for this interaction, with the P-domain serving as a secondary contact point. Notably, introducing these mutants into yeast, caused no phenotype in a wild-type background. However, when fewer origins are firing, resulting in longer stretches of leading strand synthesis before forks converge, strains expressing a Pol ε mutant lacking interaction with PCNA showed slower growth. These findings suggest that PCNA enhances the processivity of Pol ε both *in vitro* and *in vivo*.

## Graphical Abstract

**Graphical Abstract:**
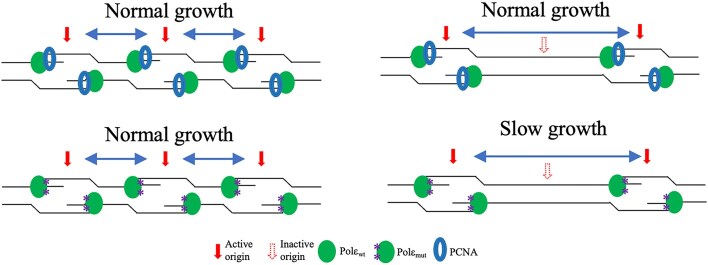

## Introduction

DNA replication in eukaryotes involves several DNA polymerases, with Pol ε and Pol δ collectively responsible for synthesizing over 90% of the DNA in a cell [1, 2]. Achieving highly processive DNA synthesis is critical for these polymerases to efficiently replicate long stretches of DNA. Many DNA polymerases enhance processivity by utilizing a clamp protein that encircles the DNA, tethering the polymerase to the template. In eukaryotes, this clamp is known as the proliferating cell nuclear antigen (PCNA), which interacts with numerous proteins involved in DNA replication and repair [3, 4].

The role of PCNA as a processivity factor for Pol ε has been debated for years, given its modest effect on Pol ε compared to the stimulation observed with Pol δ [5, 6]. Pol ε has an unusual high intrinsic processivity that makes Pol ε less dependent on PCNA [5, 6]. Initially it was shown that two small non-catalytic subunits, Dpb3 and Dpb4, contributed to the high processivity [7]. Later, the first high-resolution structure of yeast Pol ε revealed a unique processivity domain, called the P-domain that allows Pol ε to hold on to the newly synthesized duplex DNA [8], and that combined with the interaction with Cdc45–MCM–GINS (CMG) helicase raises questions whether PCNA is required to enhance the processivity of Pol ε during leading strand synthesis *in vivo* [9]. Although the P-domain in Pol ε provide a high degree of intrinsic processivity [8], limiting the need for PCNA as an external processivity factor, the role of PCNA in leading strand synthesis warrants further investigation. While *in vitro* studies have shown that Pol ε exhibits high intrinsic processivity even in the absence of PCNA [5, 10], later research demonstrated that PCNA will give a moderate stimulation of replication rates by Pol ε in a reconstituted yeast replisome system [11]. In the reconstituted human *in vitro* replication system it was observed that Pol ε is highly dependent on PCNA, but not essential, for leading strand synthesis suggesting that there are subtle differences between species [12].

The multifunctional nature of PCNA in DNA replication, repair, and chromatin assembly complicates experiments aimed at elucidating its role as a processivity factor for DNA polymerases. Thus, mutations in PCNA will not only affect the interaction with Pol ε and Pol δ, they may also affect other functions *in vivo*. For that reason, any mutations that would disrupt the interaction between Pol ε and PCNA must be engineered in Pol ε to be able to interpret the functional importance of that interaction. In this study, we aimed to dissect the functional interaction between Pol ε and PCNA. Using a combinatorial approach, including sequence–structure comparison and AlphaFold predictions of yeast four-subunit Pol epsilon bound to PCNA, we identified potential residues involved in the yeast Pol ε–PCNA interaction. We then generated a series of changes in Pol ε and analyzed their effects on DNA replication to further clarify the *in vivo* role of PCNA as a processivity factor for Pol ε.

## Materials and methods

### AlphaFold multimer prediction

Protein structure predictions were made with AlphaFold2 v2.3.2 with the AlphaFold-Multimer protocol standard parameters [13, 14], and DNA-containing predictions were made with AlphaFold3 on the AlphaFold Server [15]. The quality of predictions was assessed using the predicted local distance difference test (plDDT) [16] and template-modeling scores (pTM, ipTM) [17], where plDDT is a per-residue score, pTM an overall global score, and ipTM an interfacing residues only global score. Models with average plDDT scores ≥50 or pTM scores ≥0.5 are likely to have the correct overall fold, whereas models with average plDDT scores ≥80 or pTM scores between 0.7 and 0.8 can be considered to be of good quality [14, 16, 18]. PyMol (http://www.pymol.org) [19] was used to visualize and to superimpose structures for comparisons.

### Expression and purification of Pol ε and its mutants

Pol ε_WT_ and its mutants, namely, ΔPIP, DENK, P3, DENK/ΔPIP, and P3/ΔPIP were over-expressed in *Saccharomyces cerevisiae* strain PY116 from two plasmids, pJL1 (POL2) and pJL6 (DPB2, DPB3, and DPB4), that were under the control of Gal1-10 promoter. The expression and purification protocols were essentially similar to that described in [20]. RFC, PCNA, and RPA was purified as described in [21–23]

### Biochemical assays

#### Primer-extension assay

Reactions were performed in RQ buffer containing 40 mM Tris–HCl (pH 7.8), 100 μg/ml bovine serum albumin (BSA), and 1mM dithiothreitol (DTT). Briefly, 10 μl reaction mixture A containing 180 nM DNA (50 mer-80 mer primer-template), 8 mM magnesium acetate (MgAc_2_), and 2× physiological dNTP mix (44 μM dATP, 22 μM dGTP, 78 μM dCTP, 132 μM dTTP) in RQ buffer was mixed with 10 μl of reaction mixture B containing 8 mM magnesium acetate and 6 nM enzyme in RQ buffer. Reactions were incubated at 30°C for different time points (0, 2, 5, and 15 min), after which they were stopped by adding 20 μl of Stop solution (95% formamide, 20 mM ethylenediaminetetraacetic acid (EDTA), and 0.1% bromophenol blue). These reactions were diluted 20× in ½ Stop solution and heated at 90°C for 15 min before loading on a 10% denaturing polyacrylamide gel in 1× TBE (Tris-Borate-EDTA) buffer. The gel was scanned with Amersham Typhoon Scanner at Cy3 535 nm to excite fluorophore tetrachlorofluorescein (TET) covalently bound to 5′-end of primer.

#### Holoenzyme assay

A 15 μl reaction containing 40 mM Tris–HCl (pH 7.8), 200 μg/ml BSA, 1mM DTT, 8 mM MgAc_2_, 0.5 mM ATP, 22 μM dATP, 11 μM dGTP, 39 μM dCTP, 66 μM dTTP, α−^32^P-dCTP, 1.5 nM single-primed M13 mp18 ssDNA template, 9.2 nM four subunit Pol ε or it’s variants, 712 nM RPA, 37 nM PCNA, and 20 nM RFC, in a final concentration of 250 mM sodium acetate (NaAc) was incubated at 30°C for different time points (as indicated in Fig 5). Control reactions lacking RFC were incubated for a total of 20 min. The reactions were stopped by adding 1 μl of 0.5 M EDTA. These reactions were then purified by passing through Cytiva G-50 columns and mixed with 5 μl of loading dye (10% sucrose and 0.1% bromophenol blue) before loading them on 1% alkaline agarose gel (containing 30 mM NaOH and 2 mM EDTA). The gel was run at 30 V for 16 h, fixed in 5% tricholoroacetic acid solution for 1 h, dried at 55°C, placed on a phosphoimager screen (Fujifilm) and scanned with Amersham Typhoon Scanner.

#### Processivity assay

The substrate for processivity assay was prepared by mixing M13mp18ssDNA (NEB) with a 35-mer Oligo (5′-TET labeled) in 125 mM NaAc pH 7.8) in equimolar ratio, heating at 70°C for 5 min followed by slow cooling. The reactions were performed as described for the holoenzyme assay, but with an increased concentration of substrate (6 nM M13mp18ssDNA) and a reduced concentration of Pol ε (0.2 nM Pol ε) and RFC (5 nM RFC) to meet the single-hit conditions required in a processivity assay. The reactions (15 μl) were stopped by adding 8 μl of Stop solution (95% formamide, 20 mM EDTA and 0.1% bromophenol blue). These reactions were then heated at 90°C for 15 min. A total of 7 μl of the reaction was then loaded on 8% denaturing polyacrylamide gel electrophoresis (PAGE) in 1× TBE buffer. The gel was scanned with Amersham Typhoon Scanner. A sequencing ladder was prepared using Applied Biosystems Thermo Sequenase™ Dye Primer Manual Cycle Sequencing Kit following the manufacturer’s protocol. Three microliters of 25× diluted ddG and ddT termination reactions were loaded on the 8% denaturing PAGE.

#### *In vivo* assays

Yeast E134 strains (*ade5-1 lys2::InsEA14 trp1-289 his7-2 leu2-3 112 ura3-52*) with *P3* (KKRL736-739AAAA) and/or *ΔPIP* box (ΔQ_1193_-F_1200_) mutations in *POL2* were constructed as described in [24]. The *dpb2-202* mutation (a premature stop codon in *DPB2* truncating the last six amino acids of Dpb2) was integrated into the genome of an E134 diploid by transformation with a construct containing the *dpb2-202* mutation and a Hygromycin B resistance cassette (*hphMX*) in the same way as described in [25]. The *DPB2*/*dpb2-202-hphMX* heterozygote was sporulated and tetrads dissected to isolate a haploid *dpb2-202-hphMX* strain. As a control a hphMX cassette was introduced in the same position in a wild-type Dpb2 strain using the same procedure. The correct genotype was verified by polymerase chain reaction (PCR) and sequencing. Strains with mutation/s both in *POL2* and in *DPB2* were constructed by mating the haploid E134 *pol2* strains with the haploid E134 *dpb2-202* strain. The resulting diploids, heterozygous for *POL2*/*pol2* and *DPB2*/*dpb2-202*, were sporulated and tetrads dissected to isolate the different combinations of *pol2* and *dpb2-202* mutations in a haploid background.

### Spot dilution assay to study the impact of Sld2 degradation on PCNA interaction mutants of Pol ε

A 3xmini-AID tag, with a *kanMX* cassette linked to it, was amplified by PCR from the plasmid *pMK151* and integrated as described in [26]. The PCR product was gel purified and used to transform the E134 diploid yeast strain (*MATα/MATa ade5-1/ade5-1 lys2::InsEA14/lys2::InsEA14 trp1-289/trp1-289 his7-2/his7-2 leu2-3 112/leu2-3 112 ura3-52/ura3-52 POL2/pol2-P3,∆PIP DPB2/dpb2-202*), selecting for G418 resistance. G418 resistant clones were PCR screened for the correct integration of the 3xmini-AID tag downstream of *SLD2* with the primers SLD2-AID-conf-F, 5′-ACGGCTAAGCGAAAGGGTAG-3′ and TEF-PRO69rev 5′-GACTGTCAAGGAGGGTATTCTG-3′.

Clones with a correctly integrated 3xmini-AID tag downstream of *SLD2* were transformed with a StuI linearized pYK6 for the integration of *Gal1-10 OsTIR1* into *ura3-52* and selecting for URA^+^ clones as described in [26]. Clones with both a correctly inserted 3xmini-AID tag downstream of *SLD2* and a *Gal1-10 OsTIR1* were sporulated. The yeast strains were restreaked on Yeast extract-Peptone-Dextrose (YPD) agar plates and incubated for 2 days at 30°C. A single colony from each strain was inoculated in 50 ml of YPD media and grown overnight (15–16 h) at 30°C with shaking. Overnight cultures were pelleted and the cell pellet was resuspended in water to achieve a final OD_600_ of 1. A 5-fold dilution series was prepared. Five microliters of each dilution was plated on YP agar plates (containing 2% Raffinose + 2% galactose) with or without Indole-3-acetic acid (50 and 100 μM). The spots were allowed to dry for 10–15 min at room temperature before being incubated at 30°C for 48 h.

## Results

### AlphaFold multimer prediction model of a Pol ε–PCNA complex

**Figure 1.**
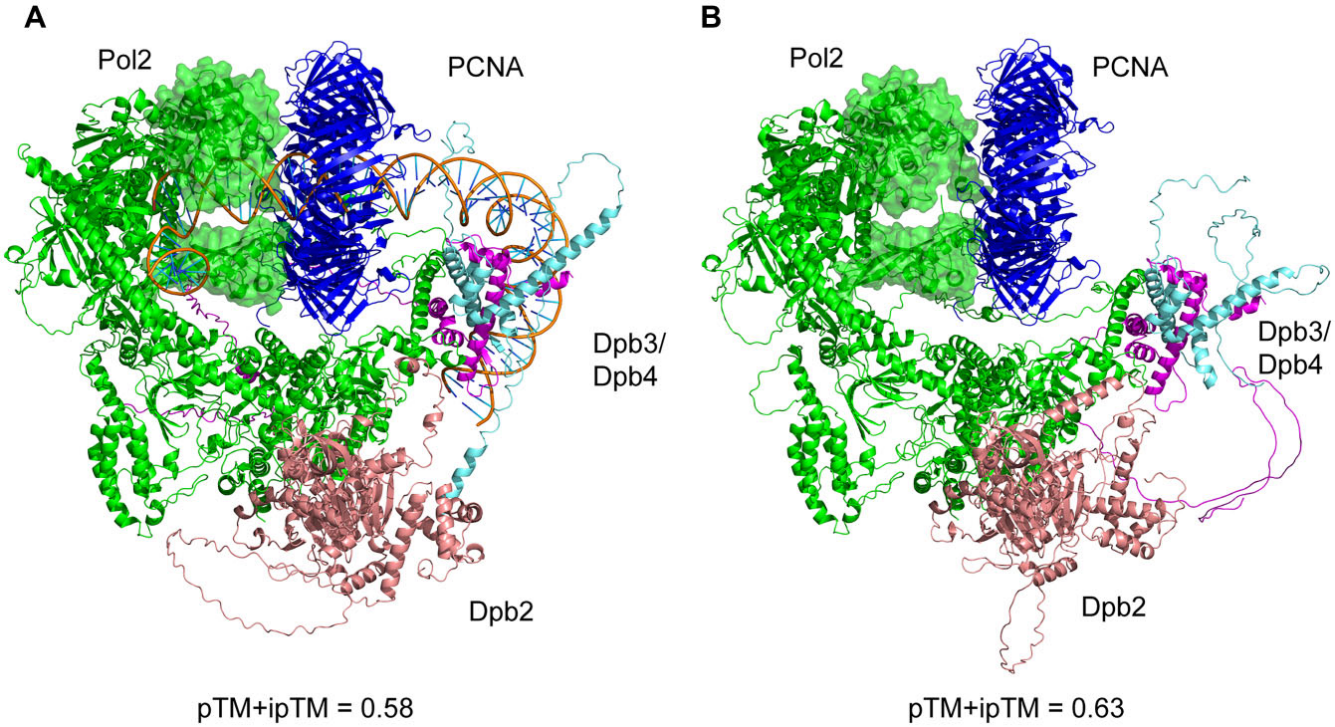
(**A**) Pol ε–PCNA–DNA interaction model predicted by AlphaFold3. (**B**) Pol ε–PCNA interaction models predicted by AlphaFold2. Pol2 is shown in green, Dpb2 in salmon, Dpb3 in magenta, Dpb4 in cyan, and PCNA in blue color. Thumb and P-domain regions that interact with DNA and are in close vicinity to PCNA are shown as shaded surface in green.

To address whether Pol ε depends on PCNA *in vivo*, we first identified residues in yeast Pol ε that are in contact with the PCNA-trimer. It was earlier suggested that a PIP-motif located in the middle of the largest subunit would be required for the interaction with PCNA [27]. However, additional interaction surfaces are also possible, as demonstrated for Pol δ [28, 29]. To identify important residues in potential interaction surfaces, we used AlphaFold 3 to model a Pol ε–PCNA–DNA complex. For comparison, we also generated a model of Pol ε–PCNA in the absence of DNA (Fig. 1). The local prediction scores for the two complexes are shown in Supplementary Fig. S2A and B. The global prediction confidence scores (pTM + ipTM) are 0.63 for the AlphaFold2 model and 0.58 for the AlphaFold3 model [13–15]. Based on these scores, both predictions are likely to have the correct overall fold. The structure predicts that PCNA is bound to the catalytic domain of Pol2 and the two smallest subunits of Pol ε, Dpb3/Dpb4, interact with the newly synthesized DNA about 40 nts away from the polymerase-active site after the DNA has passed through PCNA. The interaction between Dpb3/Dpb4 and DNA has earlier been shown to contribute to the high processivity of Pol ε and the distance correlates well with the previously reported requirement for Pol ε to achieve full processivity [7, 10]. When superpositioning the Pol ε–PCNA–DNA model with a cryogenic electron microscopy (cryo-EM) structure of Cdc45–MCM2-7–GINS–Pol ε (CMGE) (PDB ID 6hv9) [30], the position of the catalytic domain allows single-stranded DNA to be fed directly from the CMG helicase into the catalytic site of Pol ε (Supplementary Fig. S1). Minor clashes are observed between the catalytic domain of Pol ε and the CMG structure. However, considering the observed high degree of movement of the catalytic domain relative to CMG—an issue that has hindered resolution of this complex by cryo-EM [30–34]–these clashes are unlikely to cause steric problems in reality. In conclusion, the overall arrangements of Pol ε subunits in the AlphaFold model agrees well with previous experimental data.

**Figure 2.**
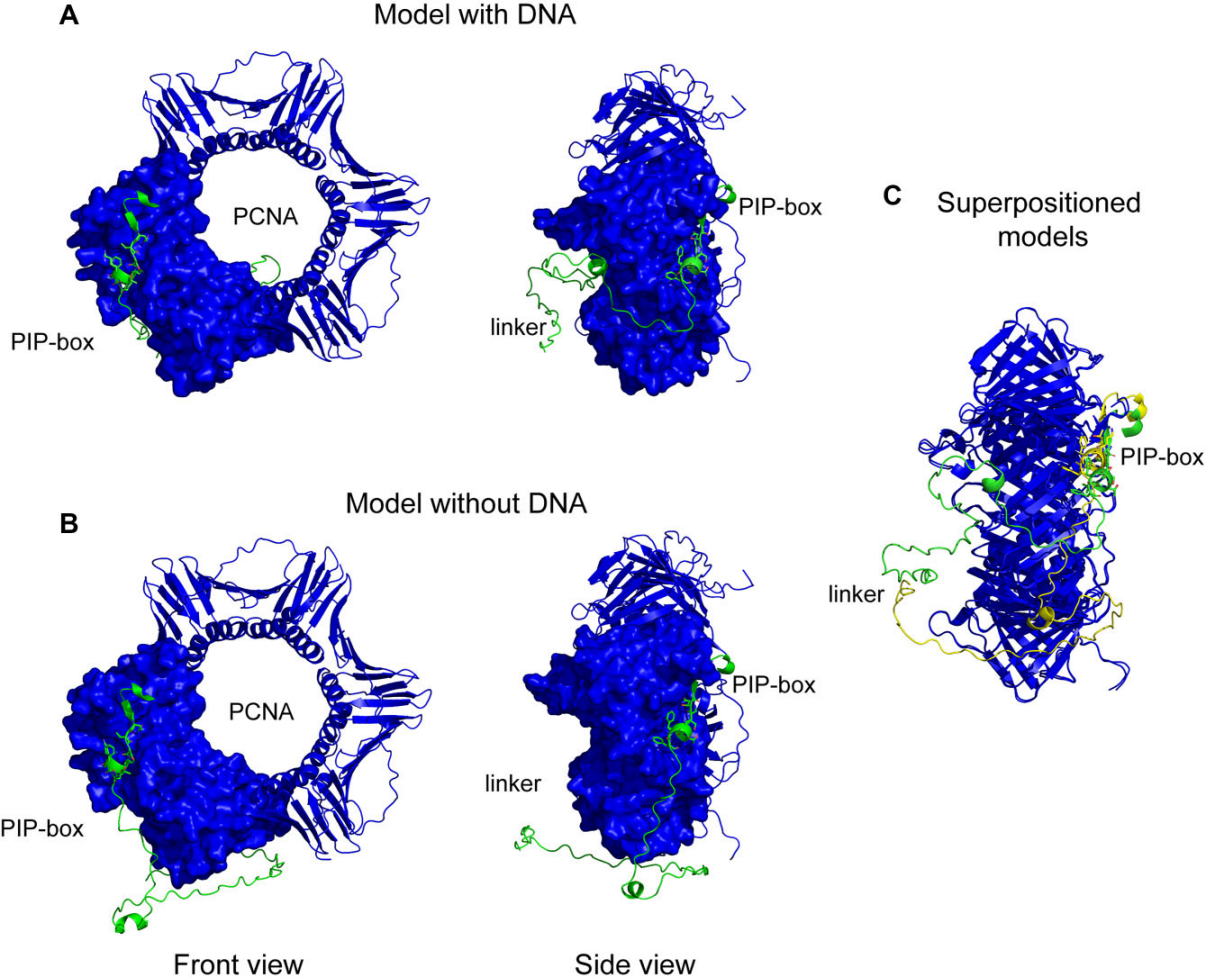
Interaction between PIP-box motif (shown as green sticks) present in the linker region and PCNA in (**A**) Pol ε-PCNA-DNA complex as predicted by AlphaFold3 and (**B**) Pol ε-PCNA complex as predicted by AlphaFold2. (**C**) The relative position of PIP-box motif (sticks) with respect to PCNA in presence or absence of DNA in the complex. The linker region and PIP-box motif is colored green for Pol ε–PCNA–DNA complex and yellow for Pol ε–PCNA complex in the superimpositioned models.

**Figure 3.**
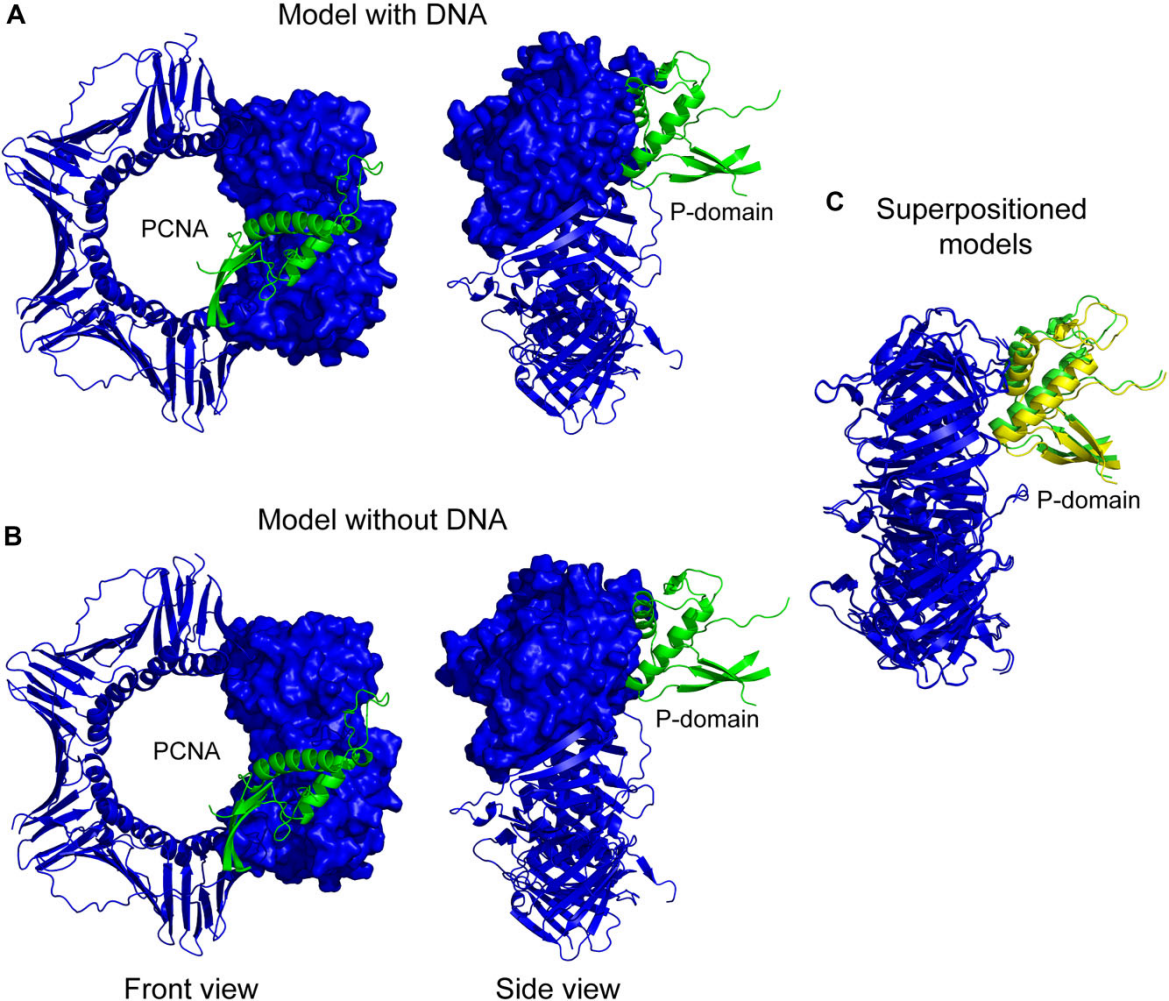
Interaction between P-domain and PCNA in (**A**) Pol ε –PCNA–DNA complex as predcited by AlphaFold3 and (**B**) Pol ε –PCNA complex as predicted by AlphaFold2. (**C**) The two models in presence and absence of DNA to view the relative position of P-domain with respect to PCNA. The P-domain is colored green for Pol ε –PCNA–DNA complex and yellow for Pol ε –PCNA complex in superimposed models.

Both AlphaFold models, with and without DNA added, suggest that Pol ε interacts with two monomers of the homo-trimeric PCNA clamp. One interaction surface involves the PIP-box motif (_1193_QTSLTKFF_1200_), and a second interaction was observed between the P-domain and another monomer in PCNA (Figs 2 and 3). The third monomer in PCNA was not found to interact with Pol ε. However, the thumb domain of the polymerase likely blocks the access of other proteins to the third monomer of PCNA. The PIP-box motif lies in a linker region that connects Pol2 N-terminal domain with the C-terminal domain. This linker region is largely disordered even when a complex is formed with PCNA and DNA, as determined by the low prediction confidence for this region in the model (Supplementary Fig. S2C).

The PIP-box interacts with PCNA through the classical hydrophobic interaction that is shared by many other proteins involved in DNA replication [3]. Specifically, Phe1200, in the PIP-box motif interacts with a hydrophobic cleft formed by two highly conserved residues, Leu126 and Ile128, located in the inter-domain connector loop of PCNA. In addition, the models suggest that several residues within the linker region are within H-bonding distance of two different PCNA monomers (Supplementary Fig. S3A and C). Gln1193, Thr1194, Ser1195, and Leu1196 in the PIP-box motif were modeled within H-bonding distance from Ala251, Pro252, and Arg44 of PCNA. It is plausible that these are functionally important interactions since it was earlier shown that pcna-90 (Pro252Ala, Lys253Ala) lost the interaction with Pol ε [35].

The second point of interaction involves the P-domain of Pol ε that interacts with a second monomer of PCNA. Two residues in the P-domain, Glu696, and Lys745 were within H-bonding distance from Lys253, Cys22, Asp41, and Asp42 of PCNA in the presence of DNA as predicted by AlphaFold3. In the absence of DNA, AlphaFold2 predicted that Asp695, Glu696, Asn698, and Lys745 in the P-domain may form H-bonds with residues in PCNA, suggesting that there may be alternative interactions between the P-domain and PCNA (Supplementary Fig. S3B and D). The residues beyond Lys253 of PCNA have low prediction confidence level, which is indicative that the interaction interface is disordered, most likely due to the location of these residues in the C-terminus of PCNA (Supplementary Fig. S2D). Keeping this in mind we also explored the interaction between Pol ε and PCNA using AlphaFold2 and found Asp695, Glu696, Asn698, and Lys745 within H-bonding distance from Lys253, Asp 256, Cys22, and Asp41 of PCNA (Supplementary Fig. S3D). Considering that the interaction between the P-domain and PCNA might rely on optional points of contacts, we decided to substitute all four residues to alanine creating the DENK mutant (Table 1). In summary, we identified two points of contact between yeast Pol ε and PCNA, the PIP-box and a less defined interaction between the P-domain and PCNA.

**Table 1.** Pol ε variants used to study interaction with PCNA

Identity	Mutation	Based upon
ΔPIP	_1193_QTSLTKFF_1200_- Deletion	Sequence conservation, AlphaFold2 and AlphaFold3 prediction
DENK	_695_D_696_E_698_N_745_K- AAAA	AlphaFold2 and AlphaFold3 predictions
P3	_736_KKRL_739_- AAAA	Structural analysis of Pol ε
DENK/ΔPIP	_695_D_696_E_698_N_745_K- AAAA + _1193_QTSLTKFF_1200_- Deletion	Sequence conservation, AlphaFold2 and AlphaFold3 prediction
P3/ΔPIP	_736_KKRL_739_- AAAA + _1193_QTSLTKFF_1200_- Deletion	Structural analysis of Pol ε, Sequence conservation, AlphaFold2 and AlphaFold3 prediction

### *In vitro* assays identify a Pol ε variant with normal polymerase activity but are not stimulated by PCNA

To construct Pol ε variants that have normal polymerase activity but are not stimulated by PCNA in *in vitro* assays, we targeted potential residues in the interface between Pol ε and PCNA. We expressed and purified wild-type Pol ε, a PIP-box deletion variant of Pol ε (ΔPIP), and two variants where amino acids were altered in the P-domain (DENK and P3 mutants) (Table 1). The DENK mutant in the P-domain was based on the two AlphaFold models as described above and the P3-mutant was designed earlier during our initial attempts to characterize the function of the P-domain. In this context the P3-mutant was selected because the substituted residues are positioned in the center of the alpha-helix that align with the nascent duplex DNA and ends with the hinge that is in proximity of PCNA when bound to DNA (Supplementary Fig. S4). Finally, we combined a deletion of the PIP-box with either the DENK or the P3 variant. All proteins were purified as four subunit Pol ε.

**Figure 4.**
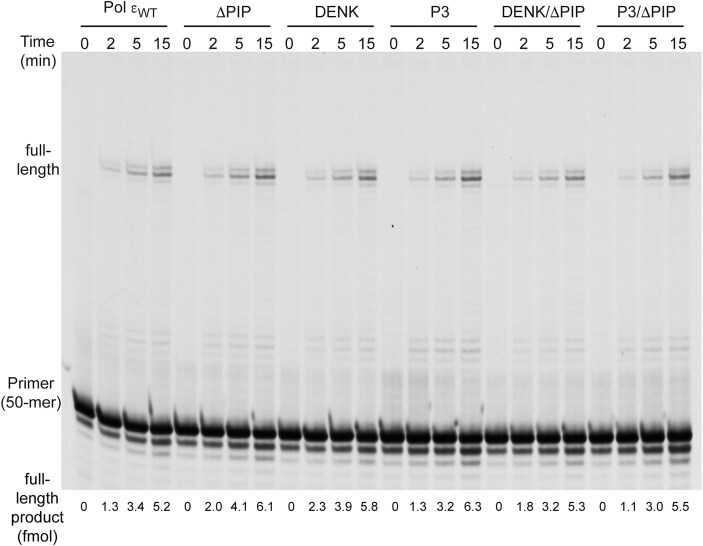
Denaturing-PAGE (10%) showing the results of a primer-extension assay with Polε _wt_ and its variants. Extension of a 5′-TET-labeled 50-mer primer annealed to an 80-mer template was studied in the presence of a physiological concentration of dNTPs [36] and at single-hit conditions. The reactions were stopped at 0, 2, 5, 15 min respectively.

**Figure 5.**
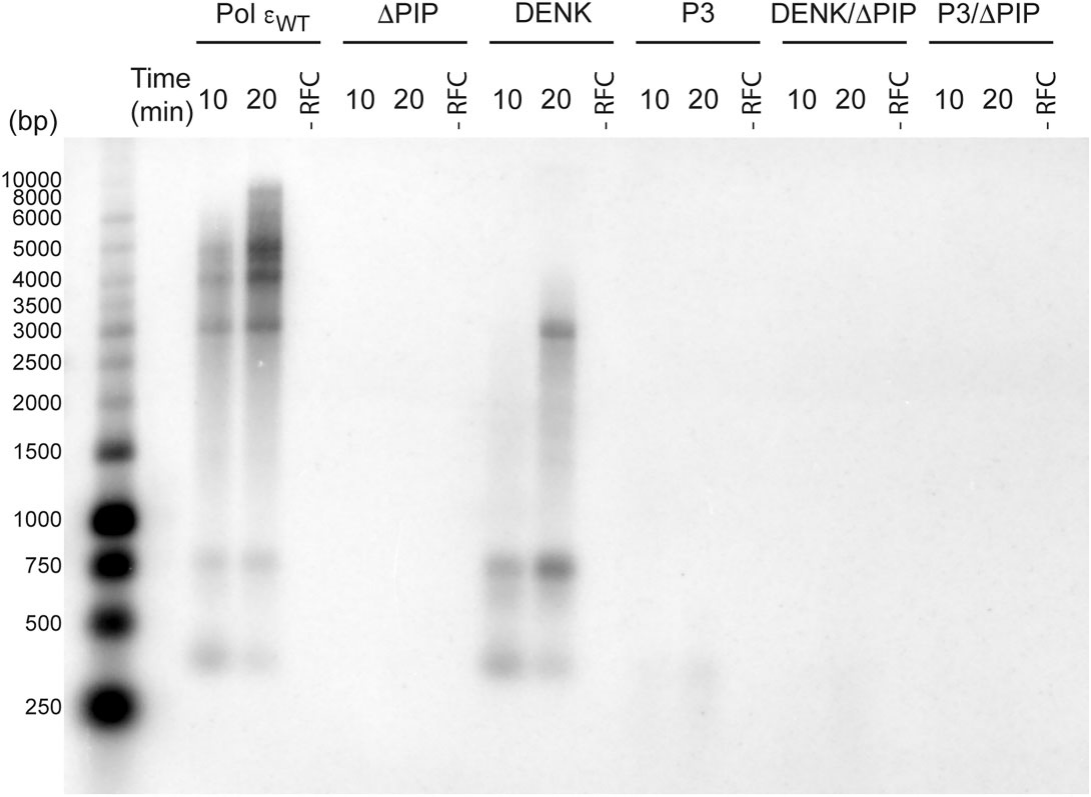
Alkaline agarose gel (1%) showing the impact of PCNA on Pol ε and its variants in the holoenzyme assay. A single-primed M13 mp18 ssDNA template (1.5 nM) was replicated by four subunit Pol ε or it’s variant (9.2 nM) in the presence of RPA (712 nM), PCNA (37 nM), and RFC (20 nM). Reactions were followed by studying the incorporation of α-P^32^-dCTP in the reaction mixtures containing 22 μM dATP, 11 μM dGTP, 66 μM dTTP, and 39 μM of dCTP [36]. Individual reactions were stopped at 10 and 20 min respectively, reactions lacking RFC was stopped at 20 min.

First, the polymerase activity of the purified Pol ε variants was compared to wild-type Pol ε in a primer extension assay, with a 50-mer primer annealed to a 80-mer template (P_50_/T_80_) (Supplementary Table S1). All variants were found to have similar polymerase activity as wild-type Pol ε under single hit conditions (Fig. 4). Next, we asked whether PCNA could stimulate all variants of Pol ε in a holoenzyme assay (Fig. 5). In this case, the ability to extend a single primer on a circular single-stranded template (M13 mp18) was explored in the absence or presence of PCNA. When compared to reactions where RFC was omitted (thus, PCNA was not loaded on the primer), Pol ε_WT_ showed an increase in DNA synthesis when PCNA was loaded by the clamp loader, RFC, onto the primer. The ΔPIP mutant showed no increase in PCNA-dependent DNA synthesis, highlighting the importance of the PIP motif for the Polε–PCNA interaction. The two P-domain mutants performed differently from each other. The DENK mutant was stimulated by PCNA, although to a lesser extent than wild-type Pol ε (reaching a stall site at 3000 bp), while the P3 mutant showed much less stimulation by PCNA (reaching about 400 bp). Still the P3 mutant was stimulated by PCNA, in contrast to the ΔPIP mutant. These results suggest that the P-domain has a functional interaction with PCNA. To ask if the PIP-box is dominant over the P-domain, we performed the same assay with a DENK/ΔPIP mutant and a P3/ΔPIP mutant. In both cases, the deletion of the PIP-box had a strong impact on the stimulation. The conclusion from these experiments is that Pol ε depends on both the PIP-box interaction with PCNA and an interaction with the P-domain in the holoenzyme assay. The observed difference between the DENK and P3 mutations may suggest that alterations in the P-domain can influence the interaction between the PIP-box and PCNA.

**Figure 6.**
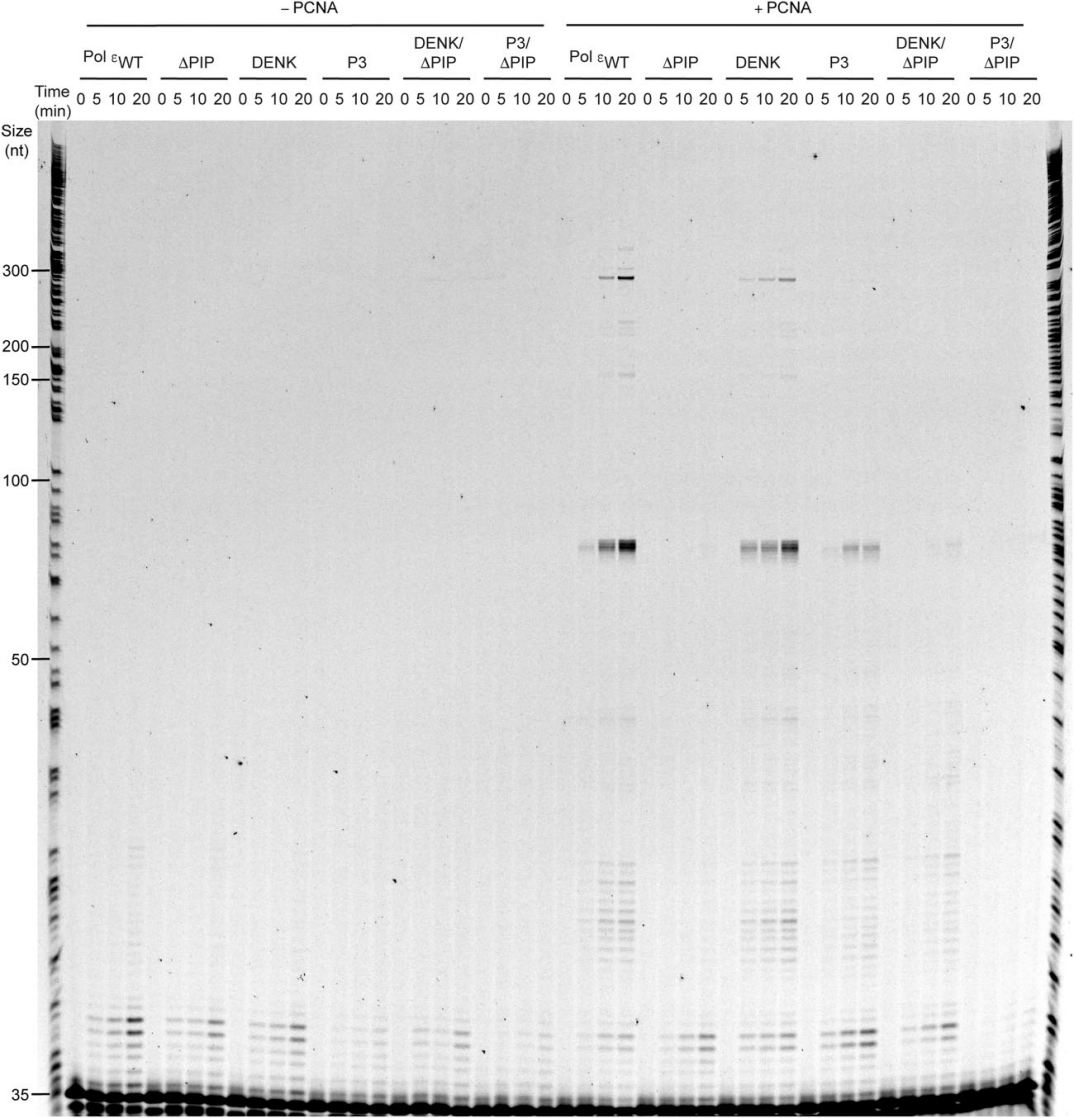
Denaturing-PAGE (8%) showing the impact of absence/presence of PCNA on processive DNA synthesis by Polε _wt_ and its variants. Extension of a 5′-TET labeled 35-mer oligo annealed to M13mp18ssDNA (NEB) was studied in the presence of 22 μM dATP, 11 μM dGTP, 66 μM dTTP, and 39 μM of dCTP [36]. Reactions were performed at 30-fold molar excess of DNA substrate over polymerase to meet single-hit criteria. Individual reactions were stopped at 0, 5, 10, and 20 min, respectively. On the far left is a sequence reaction with ddG and on the far right is a sequence reaction with ddT loaded as a size ladder, using the same template and primer as in all other lanes.

The reaction conditions above allowed multiple binding events between the polymerase and the already extended new strand. Adhering strictly to the true definition of processivity; i.e. the number of nucleotides added by polymerase during a single binding event [37], a processivity assay was carried out under single hit conditions (Fig. 6). Upon addition of PCNA, wild-type Pol ε and the DENK mutant showed a comparable increase in processivity reaching about 300 nts (Fig. 6). The P3 mutant only reached a product length of about 85 nts and the processivity of the ΔPIP mutant, was not stimulated by PCNA. When combined the DENK/ΔPIP mutant showed only a weak increase in processivity compared to ΔPIP, but the P3/ΔPIP failed to show any increase in processivity upon addition of PCNA. Clearly, the PIP-box motif seems to be the primary site of interaction between Pol ε and PCNA, irrespective of the reaction conditions used in the assays. As far as the P-domain is concerned, it seems to contribute to the interaction with PCNA since the P3 mutant with an intact PIP-box show a reduced PCNA dependent processivity. To achieve a complete loss of interaction and PCNA dependent stimulation, both the PIP-box and the P3 mutant need to be combined. Still, the results suggest that the contribution of P-domain towards PCNA binding is secondary to that of PIP-box motif in yeast.

### *In vivo* assays demonstrate a functional interaction between Pol ε and PCNA

Based on the *in vitro* results, deleting the PIP-box in Pol ε in yeast cells is expected to impact leading strand synthesis due to reduced processivity resulting from its inability to interact with PCNA. This would lead to a phenotype such as slower growth or an extended S-phase in yeast cells. However, it was earlier shown by Campbell and colleagues that the deletion of the PIP-box did not cause a growth phenotype but instead sensitized cells to treatment with the alkylating agent MMS [27]. To address this contradiction, we first wanted to confirm the earlier findings by Campbell and colleagues, and then force Pol ε to synthesize longer stretches of DNA by restricting origin firing and at the same time reduce the amount of free Pol ε in the cell. To resolve this, we used a C-terminal truncated mutation of the Dpb2 subunit of Pol ε, called Dpb2-202 (Supplementary Table S2), which affect the efficiency of origin firing most likely via reduced levels of Pol ε in the cell (see Supplementary Figs S5–S9). To restrict origin firing by lowering the concentration of a protein that is involved in the activation of origins is not a novel approach as this effect has earlier been observed for Sld2 and Sld3 in yeast [26], as well as in mouse pole4^−/−^ MEFs [38]. In our case, it was beneficial to also reduce the concentration of Pol ε and thus increase the dependence on the processivity as there are fewer Pol ε complexes that can cycle on and off the leading strand and thereby mask a reduced processivity.

**Figure 7.**
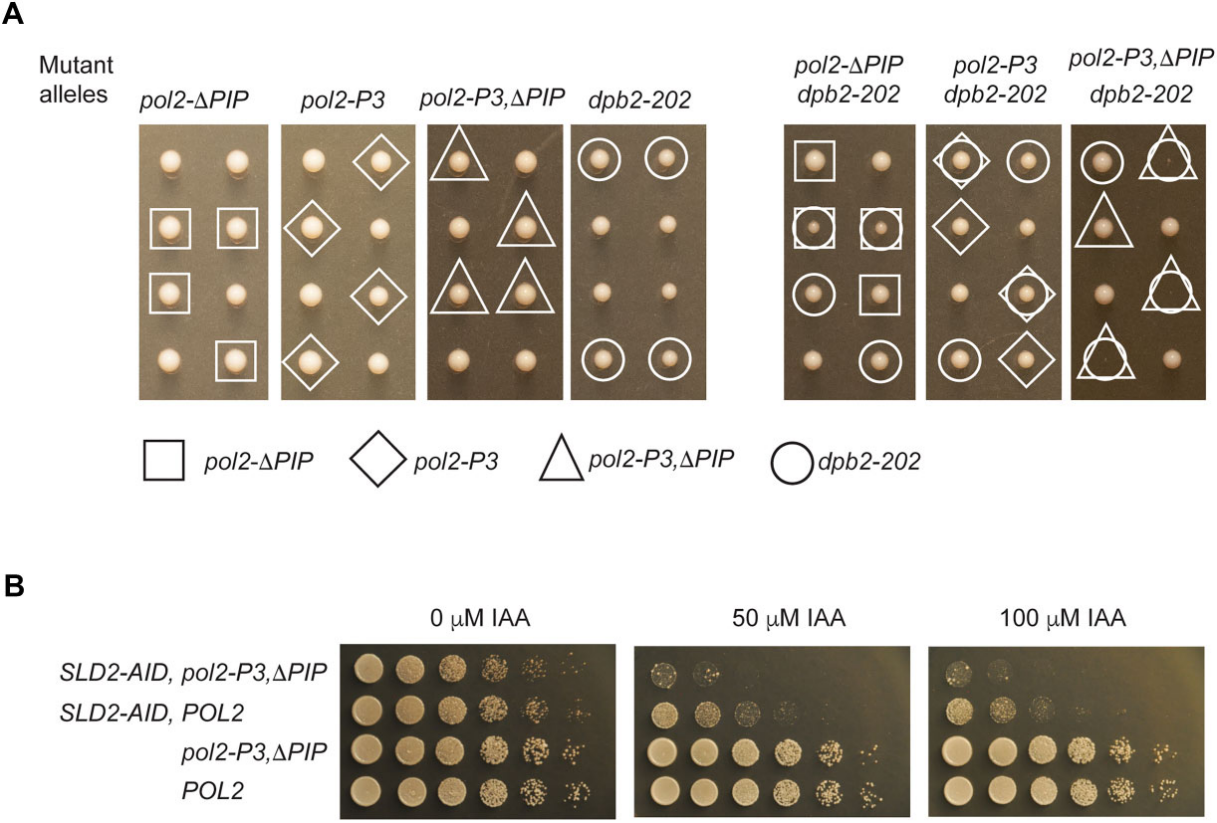
*In vivo* assays showing the impact of PCNA-interacting mutants of Pol ε on cell fitness. (**A**) Tetrad analysis of diploid yeast E134 strains heterozygous for: a single allele from left to right—*POL2/pol2-ΔPIP, POL2/pol2-P3, DPB2/dpb2-202*, and *POL2/pol2-P3,ΔPIP*, two alleles *POL2/pol2-ΔPIP DPB2/dpb2-202, POL2/pol2-P3 DPB2/dpb2-202*, and *POL2/pol2-P3,ΔPIP DPB2/dpb2-202*. Spores with the indicated mutations were grown on YPD plates for 47–50 h at 30°C. Two independent tetrads from each heterozygous strain are shown. (**B**) Spot dilution assay showing the impact of auxin induced SLD2 degradation on *SLD2-AID pol2-P3,∆PIP* and *SLD2-AID POL2* compared to *SLD2 pol2-P3,∆PIP* and *SLD2 POL2* strains. Overnight cultures were diluted to OD_600 _= 1 and followed by a five-fold serial dilution. Five microliters from each dilution was plated on YP agar plates with 2% galactose and 2% raffinose containing the indicated concentration of Indole-3-acetic acid (IAA). Plates were incubated for 2 days at 30°C.

First heterozygote diploid yeast strains in the E134 background harboring *POL2* with *pol2-ΔPIP, pol2-P3, DPB2*, or *dpb2-202* were established. After sporulation, tetrad analysis revealed that haploid spores containing *pol2-ΔPIP, pol2-P3*, or *dpb2-202* (Fig. 7A) do not cause any growth defect when compared to the colonies with wild-type Pol ε. Thus, the wild-type replication fork in the *dpb2-202* strain could support DNA synthesis over longer distance when fewer origins were activated. A deeper analysis showed that Pol ε still synthesized leading strand and that error-rates showed a near normal level, indicative that the replication fork progress normally as in wild-type cells (Supplementary Fig. S7 and Supplementary Table S3). Next, heterozygous yeast strains were constructed where we combined *dpb2-202* with either *pol2-ΔPIP* or *pol2-P3*. In this sensitized strain, a small growth defect was observed when both *dpb2-202* and *pol2-ΔPIP* was present in the same spore (Fig. 7A). In contrast, no growth defect was observed when *dpb2-202* and *pol2-P3* were combined (Fig. 7A). These results correlate well with our *in vitro* results showing that deletion of the PIP-box cause a stronger reduction in processivity. To ask if the combination of *pol2-ΔPIP* and *pol2-P3* would cause a growth defect, we placed *pol2-ΔPIP* and *pol2-P3* on the same allele in a *DPB2* or *dpb2-202* background (Fig. 7A). Again, no phenotype was observed in the *DPB2* background (*pol2- P3,ΔPIP* in Fig. 7A), but a very strong growth phenotype was observed in the *dpb2-202* background (*dpb2-202 pol2- P3,ΔPIP*) (Fig. 7A). In conclusion, these results suggest that Pol ε interacts with PCNA *in vivo* during leading strand synthesis, but the length of the leading strand synthesis in a wild-type yeast strain will not reveal a loss of interaction as other protein interactions can sufficiently support the processivity. Thus, the interaction between yeast Pol ε and PCNA does not appear to be an absolute requirement for cellular DNA replication, but when forced to replicate over slightly longer distances the dependence on the interaction between Pol ε and PCNA becomes evident.

To consolidate this conclusion, we established another yeast strain, in this case expressing Sld2 with an auxin-inducible degron (*Sld2-AID*) that allowed us to control when Sld2 should be degraded. The constructs were designed in an earlier study and it was shown that the reduction of Sld2 led to reduced origin firing efficiency. Thus, again increasing the distance between activated origins. We found that a wild-type (*POL2*) strain and a *pol2-P3,∆PIP* strain has similar growth regardless whether none, 50 μM or 100 μM IAA (auxin) was added to the agar plates. Both an *SLD2-AID POL2* strain and an *SLD2-AID pol2-P3,∆PIP* show normal growth in the absence of IAA. As expected from the study by Lynch *et al.* [26], increasing the IAA concentration to 50 and 100 μM, thus reducing the levels of Sld2 in the cells, inhibited the growth of the *SLD2-AID POL2* strain. Their conclusion was that this primarily was caused by a reduction of origin firing efficiency. Next a *SLD2-AID pol2-P3,∆PIP* strain was tested and this strain showed normal growth in the absence of IAA. However, this strain showed even a stronger reduction in growth compared to the *SLD2-AID POL2* strain when exposed to 50 or 100 μM IAA. In conclusion, we have used two different approaches to reduce origin firing efficiency, increasing the distance that leading strand synthesis need to be carried out, and in both systems a strong growth phenotype is observed with Pol ε mutants that are not stimulated by PCNA *in vitro*.

## Discussion

It is widely understood that replicative DNA polymerases which synthesize long stretches of DNA, rely on a sliding clamp to enhance their processivity. In line with this, it is undisputed that Pol δ depends on its interaction with PCNA during Okazaki fragment synthesis and other cellular processes. In contrast, Pol ε, the polymerase responsible for most of leading strand synthesis in eukaryotes, possesses a unique processivity domain (P-domain) and PCNA enhances its processivity only six-fold, significantly less than the more than 100-fold increase seen with Pol δ [5, 8]. Moreover, in a reconstituted yeast replication system, PCNA does not strongly stimulate leading strand synthesis in the presence of the helicase complex, CMG [11]. This relatively weak stimulation by PCNA has raised questions whether the observed *in vitro* effects reflect the biological conditions. In this study, we addressed this question *in vivo* and the results suggest that yeast Pol ε relies on its interaction with PCNA when leading strand synthesis extends over longer-than-usual distances.

Our AlphaFold predictions for yeast Pol ε align well with earlier biochemical findings and the recently published structures of human Pol ε (PCNA bound to the catalytic domain of Pol ε) which became available after our predictions were made [39, 40]. Notably, the human Pol ε–PCNA structures validated our AlphaFold predictions, as the models were generated before the structures were published. The AlphaFold model of yeast’s four-subunit Pol ε is of general interest, given the challenges of obtaining a high-resolution structure of DNA-bound four-subunit Pol ε due to the flexible hinge between the N-terminal and C-terminal domains of its large catalytic subunit. Previous structures have either provided a detailed view of the N-terminal domain with a poorly resolved or absent C-terminal domain, or vice versa—a well-defined C-terminal domain bound to the replicative helicase CMG, with only a shadow of the N-terminal domain [30–34]. The two small subunits in yeast, Dpb3 and Dpb4, have also been notably difficult to resolve in these structures. These subunits are known to stabilize the off-DNA rigid form of the four-subunit Pol ε, but biochemical experiments suggest they interact with newly synthesized duplex DNA, and thus enhancing the processivity of Pol ε [7]. However, the published structure of the rigid form of Pol ε does not explain how Dpb3 and Dpb4 bind to duplex DNA and stimulate processivity [41].

Interestingly, our AlphaFold predictions suggest that Dpb3 and Dpb4 have a flexible orientation, allowing them to interact with duplex DNA after it passes through the PCNA clamp. This proposed interaction site aligns well with distances determined in primer-extension assays conducted two decades ago [10]. When we superimposed the four-subunit Pol ε–PCNA–DNA model with the yeast CMGE structure, the single-stranded template leaving CMG aligned well with the entry point of Pol ε’s catalytic domain. After a sharp bend at the polymerase active site, duplex DNA exits Pol ε in a different direction, with minimal clashes between the AlphaFold model and the Cryo-EM structure of the CMGE complex. Additionally, to the best of our knowledge, the AlphaFold models are consistent with the known biochemical activities and features of Pol ε. In conclusion, while our predicted structure of the four-subunit Pol ε is likely accurate, it should be compared with actual structural data as it becomes available.

While this manuscript was being written, two independent studies published structures of human Pol ε in complex with PCNA [39, 40]. The interaction between the PIP-box and PCNA in these structures is consistent with our AlphaFold models. The interactions between the P-domain and PCNA also show partial agreement between the AlphaFold predictions and the Cryo-EM structures. A key observation from the published human Pol ε–PCNA structures is that the interaction between the P-domain and PCNA appears more flexible and most residues involved in the interaction are not conserved in yeast Pol ε, which stands in contrast to the more stable interaction between the PIP-box and PCNA [39, 40].

The *in vitro* assays with P3, DENK, and PIP-box deletion Pol ε variants led to the conclusion that the PIP-box is the primary site of interaction between Pol ε and PCNA. Deletion of this motif (ΔPIP mutant) strongly reduced the interaction between Pol ε and PCNA, which explains the lack of PCNA-dependent stimulation of DNA synthesis by Pol ε observed in holoenzyme assays (Fig. 5). However, a subtle increase in processivity upon the addition of PCNA in processivity assays for the ΔPIP mutant was observed, supporting the hypothesis that there is a functional contact between the P-domain and PCNA (Fig. 6). In contrast, four-subunit Pol ε with P-domain mutants DENK and P3 showed varying degrees of PCNA-dependent stimulation in the holoenzyme assay (Fig. 5). P3 showing much less PCNA-dependent stimulation compared to the DENK mutant. An increase in processivity upon the addition of PCNA was observed for both mutants in the processivity assay, P3 being less processive than the DENK mutant (Fig. 6). Taken together, these findings reinforce the conclusion that the PIP-box is the major site of interaction between Pol ε and PCNA and is in agreement with the conclusions drawn from similar experiments with the catalytic domain of human Pol ε [40]. However, the interaction with the P-domain is also important since the PIP-box is unaltered in the P3 mutant and still the P3 mutant show a reduction in stimulation by PCNA.

To address whether the Pol ε–PCNA interaction is important *in vivo*, we performed genetic experiments to study the effects of the ΔPIP, P3, and P3/ΔPIP mutations. The rationale was simple: if these mutations affect the PCNA-dependent processivity of Pol ε, cellular growth defects should be expected, as the leading strand synthesis would take longer to complete. Surprisingly, and in contrast to our biochemical assays, neither the ΔPIP, P3, nor P3/ΔPIP mutants displayed any growth defects (Fig. 7A). At this point, it was realized that the genetic analysis might not be sensitive enough to detect the impact of these mutations on Pol ε’s processive leading strand synthesis, considering the moderate impact that PCNA has on the processivity *in vitro* (Fig. 6 and [5, 6]). In addition to the P-domain and PCNA, the CMG helicase tethers Pol ε to the replication fork and may also contribute to its processivity. Thus, the effect of the studied Pol ε variants might be masked *in vivo*, as other factors could compensate for a loss of interaction between PCNA and Pol ε.

To explore this further, the ΔPIP and P3 variants were studied in the context of a Dpb2 C-terminal truncation mutant, *dpb2-202*, in yeast, which shows defects in origin firing efficiency and lower levels of Pol ε. Under conditions where only a few origins fire and Pol ε levels are limited, Pol ε would be required to synthesize longer stretches of DNA compared to under normal conditions. Based on this reasoning, we constructed heterozygous diploid strains with *POL2/pol2-ΔPIP, POL2/pol2-P3*, or *POL2/pol2-P3,ΔPIP* in a *DPB2/dpb2-202* background. Consistent with our *in vitro* assays, we observed that colonies with haploid cells harboring the *pol2-ΔPIP dpb2-202* mutation grow more slowly, indicating that this mutation affects processive leading strand synthesis by Pol ε. The absence of a growth defect in *pol2-P3 dpb2-202* mutant colonies further support our conclusion that the PIP-box serves as the primary site of interaction between Pol ε and PCNA, with the impact of P-domain mutations being secondary to the PIP-box deletion. Extending the genetic analysis to explore the combined effect of *pol2-P3,ΔPIP*, eliminating both known interaction surfaces with PCNA, revealed a severe growth defect (Fig. 7A). The observed gradient, where one or two interaction surfaces between Pol ε and PCNA have an increasing impact on cell proliferation in the *dpb2-202* background, supports a role for PCNA in stimulating Pol ε during leading strand synthesis *in vivo*. This was corroborated in independent yeast strains where the *pol2-P3,ΔPIP* strain showed a strong growth defect when the levels of Sld2 was reduced, leading to a reduced origin firing efficiency and longer distances for leading strand synthesis (Fig. 7B).

Based on the results of this study, we conclude that PCNA serves as a processivity factor for Pol ε *in vivo*. However, this is not a strict requirement in yeast, where the distance between origins is relatively short. The longer distances between origins in human cells may explain why the human Pol ε relies more heavily on PCNA for efficient leading strand synthesis *in vitro* [12].

## Supplementary Material

gkaf1339_Supplemental_File

## Data Availability

The data underlying this article will be shared on reasonable request to the corresponding author.
