# Supplementary material for: Evidence for a functional interaction between yeast Pol ε and PCNA in vivo
Source: Nucleic Acids Res. 2025 Dec 17;53(22):gkaf1339. doi: 10.1093/nar/gkaf1339 (PMC12709181; doi:10.1093/nar/gkaf1339)
Supplement: gkaf1339_Supplemental_File [file gkaf1339_supplemental_file.pdf]

Supplementary Material

Evidence for a functional interaction between yeast Pol ε and PCNA *in vivo*

Noopur Singh[1], Roni Odai[2], Ulf Persson[1], Göran O. Bylund[1], Ikenna Obi[1], Nasim Sabouri[1,3], Gemma C. Atkinson[2] and Erik Johansson[1]

1) Department of medical biochemistry and biophysics, Umeå University, 901 87 Umeå, Sweden
2) Department of Experimental Medical Science, Lund University, 223 62 Lund, Sweden
3) Science for Life Laboratory, Umeå University, Umeå, Sweden

Corresponding author: Erik Johansson, e-mail: erik.tm.johansson@umu.se

Table of contents:

Supplementary results ………………………………………………….…………………………3

Supplementary material and methods………………………………………………………...5

Supplementary tables 1, 2 and 3………………………………………………………..........10

Supplementary figure 1………………………………………………………………………11

Supplementary figure 2………………………………………………………………………12

Supplementary figure 3………………………………………………………………………13

Supplementary figure 4………………………………………………………………………14

Supplementary figure 5………………………………………………………………………15

Supplementary figure 6………………………………………………………………………15

Supplementary figure 7………………………………………………………………………16

Supplementary figure 8………………………………………………………………………16

Supplementary figure 9………………………………………………………………………17

Supplementary results:

Yeast Pol ε is composed of the catalytic subunit, Pol2, and three accessory subunits Dpb2, Dpb3 and Dpb4(1). Dpb2 has been shown to interact with the Psf1 subunit of GINS(2). This interaction is essential for origin activation and for the formation of the Cdc45-MCM2-7-GINS (CMG) active helicase(2). Thus, lower levels of Dpb2 in the cell would have a negative impact on origin firing.

In an earlier paper, we reported two mutations located within 13 amino acids from the Dpb2 C-terminus resulting in dpb2-201, a dpb2 protein with a proline to serine substitution and a premature stop codon truncating the protein by 6 amino acids (Supplementary table 2)(3). When combined, these two mutations in *dpb2-201* resulted in lethality when expressed in yeast and a reduced interaction between the mutant Dpb2 and (wild type) Pol2 protein was observed(3).

In this study, an allele with only the premature stop codon (no proline to serine substitution), called *dpb2-202*, was found viable but has a slower progression through S-phase compared to wild-type cells in flow cytometry experiments (Supplementary table 2 and Supplementary figure 5). However, Dpb2-202 had a similar growth rate and only slightly higher mutation rate in fluctuation assays (Supplementary figure 5 and Supplementary table 3). When purified Dpb2-202 did not have a reduced interaction with Pol2 compared to Dpb2 with GST positioned at the N-terminus of Dpb2 in pull-down experiments (Supplementary figure 6A). The Dpb2-202 protein was, however, less abundant compared to Dpb2 in cell extracts when all four subunits were over-expressed together or when expressed under its normal wild-type promoter (Supplementary figure 6B and 6C).

To determine whether the mutation in Dpb2 would destabilize Pol ε on the leading strand, thereby, allowing it to be more frequently replaced by another polymerase, we combined *pol2-M644G* and *rnh201Δ* with either *dpb2-202* or *DPB2* and studied the incorporation of ribonucleotides into the nascent leading strand in these two yeast strains using Southern Blot (Supplementary figure 7). We found that the incorporation of ribonucleotides by Pol ε into the nascent leading strand was similar in both strains indicating that Pol ε is also the primary polymerase on the leading strand in Dpb2-202 cells. Thus, the minor increase in mutation rate is not explained by Pol ε having lost its role as the primary leading strand polymerase.

To test the possibility that *dpb2-202* could affect the efficiency of origin firing, we synchronized *E134 MATa Dpb2-202* and *DPB2* strains in G1 with alpha factor and released them into media containing 200mM hydroxyurea. Since hydroxyurea does not inhibit the firing of early origins but slows down the replication fork progression(4), it allowed us to study the length of replication intermediates from early origins as the synchronized cells slowly progressed through S phase. Genomic DNA was isolated from cells collected during G1 synchronization and after release into the HU containing media at 30, 60 and 90 min (Supplementary figure 8). The nascent DNA was separated from the un-replicated DNA on an alkaline agarose gel and analysed with southern blot using probes specific for the early origins ARS305 and ARS306, and the late origin ARS522. Replication intermediates from the early firing origins in the wild-type strain was detected in the southern blot after 30 min in HU and the molecular weight of the nascent DNA fragments increased gradually after 60 and 90 min in a similar way observed in previous studies (4,5). Similar to the wild-type, the detection of the early origin replication intermediates from *dpb2-202* genomic DNA was also detected after 30 min in HU with an increase in the size of the fragments after 60 and 90 min. However, the intensity of the replication intermediates was lower and the molecular weights of the fragments were slightly higher. This suggests that the origin firing of ARS306 and ARS305 is less efficient

in the dpb2-202 strain compared to the wild type strain. However, we did not detect replication intermediates from the late origin ARS522 which is expected, since the firing of late origins is repressed in the presence of HU.
Considering that fewer origins were active in the dpb2-202 strain, the distance between origins would increase and at the same time leading strand synthesis would be carried out for a longer distance in such cells. To ask whether activated origins are located further apart, we established a yeast strain that was able to incorporate BrdU when replicating DNA. Cells from wild type and dpb2-202 strains were synchronized using alpha-factor, then released and harvested after 110 minutes, followed by a DNA fiber analysis such that we could visualize the position of origins and determine the distance between origins. The median distance between two origins increased in dpb2-202 cells, when compared to the wild-type cells (Supplementary figure 9).

In conclusion, the dpb2-202 mutant results in lower levels of four-subunit Pol ε, but Pol ε dpb2-202 still remains the dominant polymerase during leading strand synthesis. However, the number of activated origins is restricted due to the lower levels of four-subunit Pol ε in the dpb2-202 strain. Thus, the cells harbouring dpb2-202 mutation will be highly sensitive to changes that lead to reduced processivity of Pol ε due to the increased inter-origin-distance and the lower levels of free Pol ε in dpb2-202 cells. This presents an interesting opportunity where we can use the dpb2-202 background to monitor changes in processivity of Pol ε that would otherwise not be apparent in wild-type cells.

Supplementary Materials and Methods

Construction of strains

Yeast E134 strains (*ade5-1 lys2::InsEA14 trp1-289 his7-2 leu2-3,112 ura3-52*) with *P3* (KKRL736-739AAAA) and/or *ΔPIP* box (ΔQ_{1193}-F_{1200}) mutations in *POL2* were constructed as described in (6). POL2 mutations were confirmed by PCR using primers- Screen ΔPIP 5'- GAG GAT AAG TTT AAG TCG AAG-3' with Pol2 3933R 5'-CCT AAT CAT ACT TCC TAG TGC AC-3' and Screen W2-3 5'- GTT ATC CAC ATA GCT GCA GCT GC-3' with Pol2-26 5'-ATT AGT TGT AAT TCA CCA CGC CT- 3'

The *dpb2-202* mutation (a premature stop codon in *DPB2* truncating the last 6 amino acids of Dpb2) was integrated into the genome of an E134 diploid by transformation with a construct containing the *dpb2-202* mutation and a Hygromycin B resistance cassette (*hphMX*) in the same way as described in Isoz *et al.*, 2012. The *DPB2*/*dpb2-202-hphMX* heterozygote was sporulated and tetrads dissected to isolate a haploid *dpb2-202-hphMX* strain. The correct genotype was verified by PCR and sequencing.

Double mutant strains with mutation/s both in *POL2* and in *DPB2* were constructed by mating the haploid E134 *pol2* strains with the haploid E134 *dpb2-202* strain. The resulting diploids, heterozygous for *POL2*/*pol2* and *DPB2*/*dpb2-202*, were sporulated and tetrads dissected to isolate the different combinations of *pol2* and *dpb2-202* mutations in a haploid background.

Cell cycle analysis using flow cytometry

The cells were grown at 30°C in YPD (yeast extract 1%, peptone 2% and glucose 2%) from OD 0.05 to logarithmic phase at OD 0.3. Cultures (4ml) with OD 0.3 were pelleted and the cells were fixed by resuspending the pellet in 5ml 70% ethanol and storing it overnight at 5°C. The cells were washed in 750µl H_2O, resuspended in 0.5ml RNaseA solution (boiled 2mg/ml RNaseA in 50mM Tris pH 8.0 and 15mM NaCl) and incubated at 37°C for 12-15 hours. Fifty microliters of Proteinase K (20mg/ml in H_20) was added to the cell samples and it was incubated at 50°C for 1h 20min. The cells were centrifuged and resuspended in 0.5ml 50mM Tris-HCl pH 7.5. Fifty microliters of the cell suspension was added to 1ml 50mM Tris-HCl pH 7.5 with 1x SYBR Green (Invitrogen cat.no.S7563) to stain the genomic DNA. The samples were sonicated at the lowest output for 10 sec and the stained DNA of the cells was measured in a FC Cytomics 500 Flow cytometer from Beckman Coulter Inc.

Synchronization of yeast cells

The cells were grown to logarithmic phase in 30°C YPD media as described above. Alpha factor (Genscript cat.no. GENSRP01002) was added to the cells at OD 0.3 to a concentration of 5µg/ml to arrest the cells in the G1-phase. After 75min, the same amount of alpha factor was added again. At 110 min after addition of alpha factor, the cells were washed by centrifugation and resuspension in an equal volume of cold H_2O. The cells were centrifuged again, resuspended in 1ml cold H_2O and transferred to a new tube. Cold water of the same volume as the initial culture was added and the cells were centrifuged again. The cells were synchronously released into the S-phase by resuspending the pellet in pre-warmed 30°C YPD media.

Determination of *in vivo* levels of Dpb2

Starter cultures of *Saccharomyces cerevisiae* E134 *DPB2* and *dpb2-202* strains grown in YPD media overnight, were diluted into 50ml fresh pre-warmed YPD to an OD_{600} of 0.025. Cell growth was monitored until an OD_{600} of about 0.7, after which the cells were harvested and

washed once in 1ml of water before being frozen in liquid nitrogen. Cell pellets were stored at -80°C until further use.
Cell extracts were prepared by resuspending cell pellets in a cracking buffer: 8M urea, 5% SDS (w/v), 40mM Tris-HCl pH=6.8, 0.1mM EDTA, 0.4mg/ml bromophenol blue, 15mM β-mercaptoethanol and one tablet protease inhibitor cocktail (Roche Diagnostics) per 10ml cracking buffer, followed by boiling for 5 minutes. Insoluble material was pelleted at 37547xg for 15 minutes at 20°C and the supernatants were transferred to fresh tubes, diluted five times in cracking buffer before 10µl was loaded on a 10% polyacrylamide gel. Half of the samples were spiked with 20ng of purified recombinant four-subunit DNA polymerase ε in order to verify the presence of Dpb2 in the following western blot. Gels were run at 125V for 1.5 hours and protein was transferred to Amersham Hybond P 0.45 PVDF membranes (Cytiva) at 25V, 130mA for 1.5 hours in a Trans-blot semi-dry transfer cell (BIO-RAD). Membranes were blocked in blocking buffer, 25mM Tris-HCl pH=8.0, 150mM NaCl, 5% milk and 0.3% Tween-20 (w/v) for two hours at room temperature, washed thrice for 5 minutes in a washing buffer (same as blocking buffer, but lacking milk) and thereafter incubated with a rabbit α-Dpb2 antiserum in washing buffer supplemented with 2% milk for 2-3 days at 5°C. Membranes were washed in washing buffer thrice for 10 minutes at room temperature followed by incubating them with a horse radish peroxidase conjugated α-rabbit secondary antibody (NA934V, Cytiva), diluted to 1:10000 in washing buffer supplemented with 2% milk, at room temperature for one hour. Membranes were again washed thrice for 10 minutes in washing buffer before developing with Amersham ECL Prime western blotting detection reagents (Cytiva) and ECL signals were detected by exposing Amersham high performance chemiluminescence film (Cytiva) to the membranes.

Over-expression and purification of recombinant GST-tagged Polε with/without the mutant Dpb2-202 protein.
The protease deficient yeast strain PY116 (*ura3–52, trp1-289, his3–11, 15, pep4–4, prb1–1122 (prc1–407) (CAN^s), leu2–3, 112, nuc 1: LEU2*) was transformed with the plasmids pJL1 (expressing Pol2 with/without a GST-tag), pJL5 (expressing Dpb3 and Dpb4) and pJL9 (expressing either wild type Dpb2 or mutant Dpb2-202 with or without a GST-tag) and all genes were under the control of the galactose inducible promoter *Gal1-10* (7). Cells were grown in 2.4 liters of selective glycerol lactate media and at an OD_{600} of 1 to 2 an equal volume of supplemental media was added. Cell growth was continued for about 4 hours followed by the induction of protein expression by the addition of powdered galactose to a final concentration of 2% (w/v). Protein was expressed for 4 hours and thereafter cells were harvested and frozen in liquid nitrogen. A cell powder from the frozen cells was produced by grinding the cells under liquid nitrogen in a SPEX CertiPrep 6850 Freezer/Mill. The cell powder was stored at -80°C until use. Cell extracts were prepared by thawing the cell powder in an equal volume in milliliter of 2x lysis buffer (300mM Tris-Acetate pH=7.8, 100mM Na-Acetate, 4mM EDTA, 2mM EGTA, 20mM $NaHSO_3$, 2mM DTT, 10µM Pepstatin A, 10µM Leupeptin, 10mM Benzamidine and 2mM PMSF) as grams of cells. From now on all steps were performed at 4°C. Saturated 4M ammonium sulfate was added to a final concentration of 175mM and in order to precipitate the DNA a 10% polymin P solution (40µl per ml of extract) was added. The extract was incubated for 15 minutes with occasional stirring followed by centrifugation at 35000xg for 1 hour. Protein was precipitated by slowly adding 0.28 grams of powdered ammonium sulfate per ml extract while stirring. When all ammonium sulfate had gone into solution stirring was continued for another 45 minutes. Protein was pelleted at 41000xg and slowly dissolved in a B_0 buffer (25mM HEPES pH=7.6, 10% glycerol, 0.05% NP-40, 1mM EDTA, 0.5mM EGTA, 1mM DTT, 2µM Pepstatin A and 2µM Leupeptin). The

dissolved protein was frozen in liquid nitrogen and stored at -80°C. The subscript (n) in B(n) Buffers denote the concentration of sodium acetate in mM. Next the frozen protein extract was slowly thawed and dialyzed against 200ml B_0 for 40 minutes and 200ml B_{50} for 40 minutes. If needed the conductivity of the dialyzed protein extract was adjusted to the same conductivity as B_{300} with B_0. In order to remove precipitated protein the extract was centrifuged for 30 minutes at 41000xg before loading it on a phosphocellulose column (10ml bed volume) equilibrated with B_{300}. The column was washed with B_{300} and protein eluted with B_{700}. Protein containing fractions were identified by Bradford analysis and pooled. The pooled protein fractions were diluted to B_{300} with B_0 and 1ml of Glutathione Sepharose 4B (equilibrated in B_{300}) was added. Protein was batch bound overnight at a rotation speed of 5rpm. The Glutathione Sepharose was washed with B_{300} and protein was eluted with B_{300} supplemented with 20mM reduced glutathione (pH adjusted to 8.0). Finally, the protein preparations were run on a miniSuperose 6 column equilibrated in B_{400}.

Determination of levels recombinantly expressed wild type Dpb2 and mutant Dpb2-202 by western blot.

The strain PY116 was transformed with the plasmids pJL1-GST-POL2, pJL5 and pJL9 (expressing Dpb2 or Dpb2-202) and grown in the same way as above for expression of Polε. Cells, before and after induction of expression, were pelleted and dissolved in SDS-containing loading buffer, boiled for 5 minutes and loaded on a 10% polyacrylamide gel. Gel running and western blot was done as described above for determination of the *in vivo* levels of native Dpb2 with one exception. The western blot membrane was only incubated overnight with the rabbit α-Dpb2 antiserum.

Measurement of mutation rates

The spontaneous reversion rate in *lys2::InsE-A$_{14}$* and *his7-2* and forward mutations in *CAN1* of the E134 Dpb2 and Dpb2-202 strains were measured using fluctuation assays.

Two independent fluctuation assay experiments were performed with nine cultures from each strain for a total of 18 cultures per strain. Both experiments were performed using the same Dpb2 and Dpb2-202 strain isolates. The cultures were grown from two day old colonies on YPD plates to stationary phase in 5ml 30°C YPD over 2 days and processed as described in (8,9) with the exception that the SC-Arg + Can plates contained 60µg/ml L-canavanine in the Can^R mutant count and SC plates were used for the viable count. The plates were counted after 2-3 days of incubation at 30°C and the mutation rate was calculated from the mutation frequencies as described previously(8,9).

Detection of ribonucleotides in genomic DNA with single-stranded probes

The yeast strains were grown in YPD at 30°C to OD 1 and genomic DNA was isolated using Epicenter MasterPure Yeast DNA purification kit (cat.no. MPY80200). The concentration of DNA was measured using Qubit dsDNA BR Assay Kit (cat.no. Q32850). The genomic DNA was subjected to alkaline hydrolysis with 0.3M KOH for 2h at 55°C and mixed with 6x alkaline loading buffer (300mM KOH, 9mM Tris-HCL, 0.135% orange G, 0.027% xylene cyanol FF, 54% glycerol and 54mM EDTA). DNA was separated on an alkaline agarose gel (1% Agarose, 50mM NaOH, 1mM EDTA) for 22-25h at 25V. The amount of DNA loaded on the gel was 200ng for the *pol2-M644G rnh201Δ* strains with *DPB2* or *dpb2-202* and 430ng for the *pol3-L612M rnh201Δ* strains with *DPB2* or *dpb2-202*. The gel was neutralized with 0.1M Tris pH 7.5, stained with GelRed (Biotium cat.no. 41003) and photographed to determine the positions of the bands in the DNA ladder. The gel was cross-linked at 1200J/m^2 in a CL-1000 Ultraviolet cross-linker and treated with denaturation solution (0.5M NaOH, 1.5M NaCl) and blotting solution (0.25M NaOH, 1.5M NaCl). The DNA was transferred to a Hybond-XL nylon

membrane (RPN203S) by capillary action over-night. Southern analysis was performed using single stranded radiolabeled probes created using a PCR amplified 670bp dsDNA template from the FUS1 gene 1.5kb upstream of origin ARS306. Three primers at a distance of 200bp from each other were used together with Megaprime DNA labeling system (GE Healthcare cat.no. RPN1604) and α-^{32}P labeled dCTP (6000Ci/mmol, 20mMCi/ml) to make each probe. The labeled probe was purified with Illustra ProbeQuant G-50 micro columns (cat.no. 28-9034-08). After hybridization the nylon membrane was washed, placed on a phosphoimager screen and scanned on a Typhoon 9400 phosphoimager (GE Healthcare).

The Pol ε ribonucleotide incorporating strains with *DPB2* or *dpb2-202* in combination with *pol2-M644G* and *rnh201Δ* were created by introducing *pol2-M644G* into the genome of the heterozygous E134 Dpb2 and Dpb2-202 strains via integration-excision using a p173-*pol2-M644G* plasmid (McElhinny et al. 2010 gift from Tom Kunkel). The *RNH201* gene was deleted by transformation with a PCR construct containing a KanMX cassette flanked by 45bp of sequence homologous to the upstream and downstream regions of *RNH201*. The correct insertion of *pol2-M644G* and deletion of *RNH201* was confirmed by PCR and sequencing. The heterozygous strain was sporulated and tetrad dissected to obtain a haploid.
The Pol δ ribonucleotide incorporating strains with *DPB2* or *dpb2-202* in combination with *pol3-L612M* and *rnh201Δ* were created by mating the E134 *MATα* Dpb2 and Dpb2-202 strains with JSW11, a *MATa pol3-L612M rnh201Δ* strain in a Δ|(-2)|-7B-YUNI300 background (Lujan et al 2013). The diploid was sporulated and tetrad dissected to obtain a haploid and the genotype was confirmed with PCR.

Detection of replication intermediates in genomic DNA
Cultures of Dpb2 and Dpb2-202 Mat a strains were grown to logarithmic phase in YPD at 30°C, arrested in the G1 phase with alpha factor and washed with cold H_2O as described above. The cell pellet was resuspended in 25°C YPD media with 200mM hydroxyurea and incubated at 25°C while shaking. Samples for genomic DNA isolation and flow cytometry cell cycle analysis were taken before the release from the G1 cell cycle block and after the addition of hydroxyurea at 30, 60 and 90min. The genomic DNA was isolated and measured as described above. To separate the replication intermediates from unreplicated genomic DNA, 1µg of the DNA was mixed with 6x loading dye and separated on a 1% alkaline agarose gel and analyzed by Southern blot. The radiolabeled probes specific for ARS305, ARS306 and ARS522 were created by using a PCR amplified 1 kb region containing the origins as a template with Amersham Rediprime II Random Prime Labelling System (RPN1633) and α-^{32}P labeled dCTP.

***In vivo* labelling of DNA for DNA fiber analysis**
Construction of strains; The two haploid strains 2320 (*MATa, URA3::GPD-TK(5*x*), AUR1c::ADH-hENT1, RAD5+, Δbar1*) and 2324 (*MATα, ura3, AUR1c::ADH-hENT1, RAD5+, Δbar1*) (a kind gift from Philippe Pasero) were mated and the resulting diploid was sporulated. Tetrads were dissected to isolate two new haploid strains with the same genotype (*URA3::GPD-TK(5*x*), AUR1c::ADH-hENT1, RAD5+, Δbar1*), except having different mating types. The two new strains were mated and the resulting diploid was transformed with a PCR product covering the *dpb2-202* mutation and the *hph kanMX* cassette with flanking sequences. The *URA3::GPD-TK(5*x*), AUR1c::ADH-hENT1, RAD5+, Δbar1*, *DPB2/dpb2-202 hph kanMX* diploid was sporulated and by tetrad dissection two haploid strains (*MATa, URA3::GPD-TK(5*x*), AUR1c::ADH-hENT1, RAD5+, Δbar1, DPB2* and *MATa, URA3::GPD-TK(5*x*), AUR1c::ADH-hENT1, RAD5+, Δbar1, dpb2-202 hph kanMX)* from the same tetrad, were isolated and used for *in vivo* labelling of DNA with BrdU as described below.

In vivo labelling of DNA with BrdU; Overnight grown cultures were diluted to OD_{600}=0.025 in fresh pre warmed YPDA media and grown to OD_{600}=0.3. At t=0 minutes a sample was withdrawn from the cultures, for flow cytometry and to the rest alpha-factor was added to a final concentration of 5µg/ml to arrest the cells in the G1 phase. At t=70 minutes alpha-factor was again added to a final concentration of 5µg/ml. At t=95 minutes BrdU was added to the cultures to a final concentration of 40µg/ml and at t=110 minutes cells were washed in water and released into fresh pre warmed YPDA media supplemented with hydroxyurea to a final concentration of 200mM. 90 minutes after the release BrdU labelling was stopped by mixing 700µl of the cells with 350µl stop solution (0.5M EDTA, 0.3% Na-azide w/v). The cells were pelleted at 1500xg, resuspended in 500µl 70% ethanol and stored at 4ºC until used for DNA combing. Samples for flow cytometry were taken at t=110 minutes and at 90 minutes after the release.

DNA fiber analysis
Spreading of DNA fibers on glass slides was performed as previously described in Obi et al. (10). Briefly, yeast cells treated with 200U/ml lyticase from *Arthrobacter luteus* (Sigma-Aldrich) to digest their cell wall were placed on a microscope slide (Superfrost Plus, Thermo Fisher Scientific) and lysed using lysis solution (50mM Tris-HCl (pH 7.4), 25mM EDTA, 500mM NaCl, 0.1% Nonidet *P*-40, 1% sodium dodecyl sulfate (SDS), and 5mM β-mercaptoethanol). DNA was stretched along the slide tilted to 15°, fixed with 4% paraformaldehyde and subsequently denatured with 0.5M NaOH. BrdU incorporated into DNA was detected using rat anti-BrdU clone [BU1/75 (ICRI)] primary antibody (ABD Serotec) and goat anti-rat IgG Alexa Fluor 568 secondary antibody (Life Technologies), while ssDNA was detected using anti-DNA antibody, single-stranded, clone 16–19 primary antibody (Sigma Aldrich) and goat anti-mouse IgG2a (γ2a) Alexa Fluor 488 secondary antibody (Life Technologies). DNA fibers were visualized using Axio Imager Z1 microscope (Zeiss) and images of untangled DNA fibers were obtained at randomly selected fields of view. Only DNA fibers with BrdU labeling with intact ssDNA ends were selected for analysis using the Zen 2.6 blue edition (Zeiss).

Supplementary Tables

Supplementary Table 1. Sequence of oligonucleotides

Name	Length	Sequence 5'-3'
Oligo-M13	35	TET-CCCAGTCACGACGTTGTAAAACGACGGCCAGTGCC
50mer	50	TET-GATCAGACTGTCCTTAGAGGATACTCGCTCGCAGCCGTCCACTCAACTCA
80 mer	80	CAGCTTGATAGTCAGTGACGTTGTTCTGGATGAGTTGAGTGGACGGCTGCGAGCGAGTATCCTCTAAGGACAGTCTGATC

Supplementary Table 2. Amino acid sequence of Dpb2 variants

Dpb2	671-RRARYMEYVP SSKKTIQEEI YI* -692
Dpb2-201	671-RRARYMEYV**S** SSKKTI **-686
Dpb2-202	671-RRARYMEYV**P** SSKKTI **-686

Amino acid substitutions and stop codons (*) are in bold.

Supplementary Table 3. Spontaneous mutation rates as observed in fluctuation assay

**Strain**	**Mutation Rate (x 10^{-8}) * (95% confidence limits)**					
	**Lys^{+}**		**His^{+}**		**Can^{r}**	
	Absolute rate	Relative rate (mut vs wt)	Absolute rate	Relative rate (mut vs wt)	Absolute rate	Relative rate (mut vs wt)
DPB2	19.4 (16.3-22.2)	1	1.65 (1.28-2.19)	1	68.6 (62.3-84.6)	1
dpb2-202	43.1 (34.4-52.0)	2.22	3.29 (1.80-4.29)	1.99	112 (93.0-141)	1.63

Mutation rates are given as median of 18 independent cultures.

Supplementary Figures

Supplementary figure 1

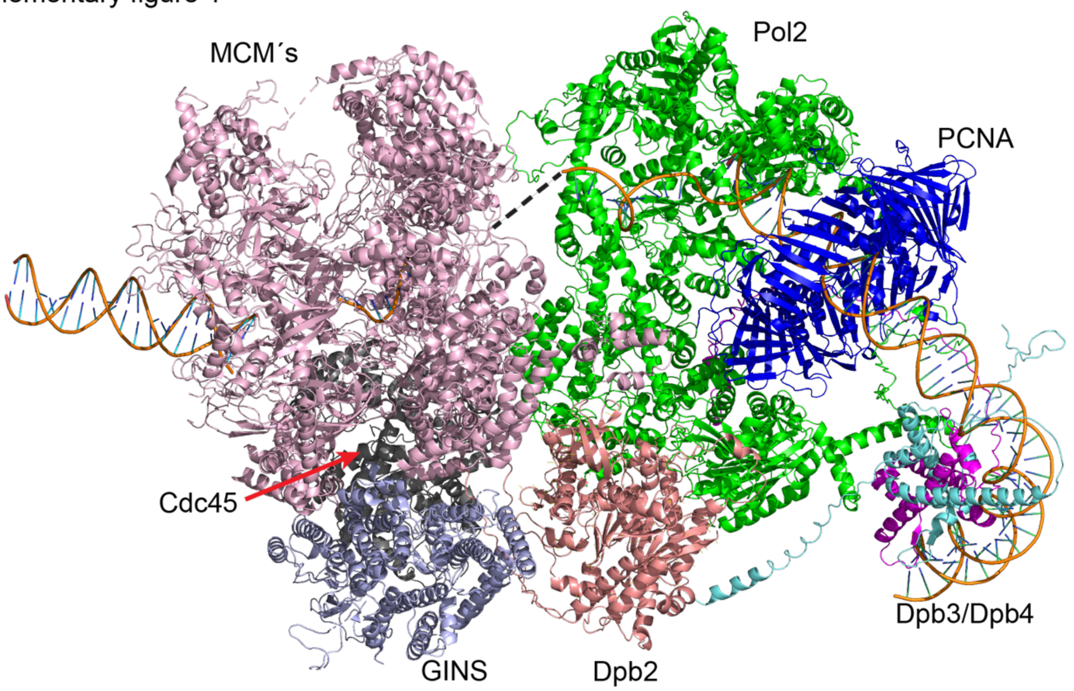

Supplementary Figure 1. A Pol ε -PCNA-DNA complex predicted by AlphaFold 3 aligned with a CMG-Pol ε complex (PDB ID- 6HV9) with single-stranded DNA exiting from the CMG-helicase in the direction of the polymerase site in Pol ε. Pol2 is shown in green, Dpb2 in salmon, Dpb3 in magenta, Dpb4 in cyan,PCNA in blue color, Mcm2-7 in light pink, GINS in ice blue, Cdc45 in gray, and the trajectory of the ssDNA is drawn as a dashed line.

Supplementary figure 2

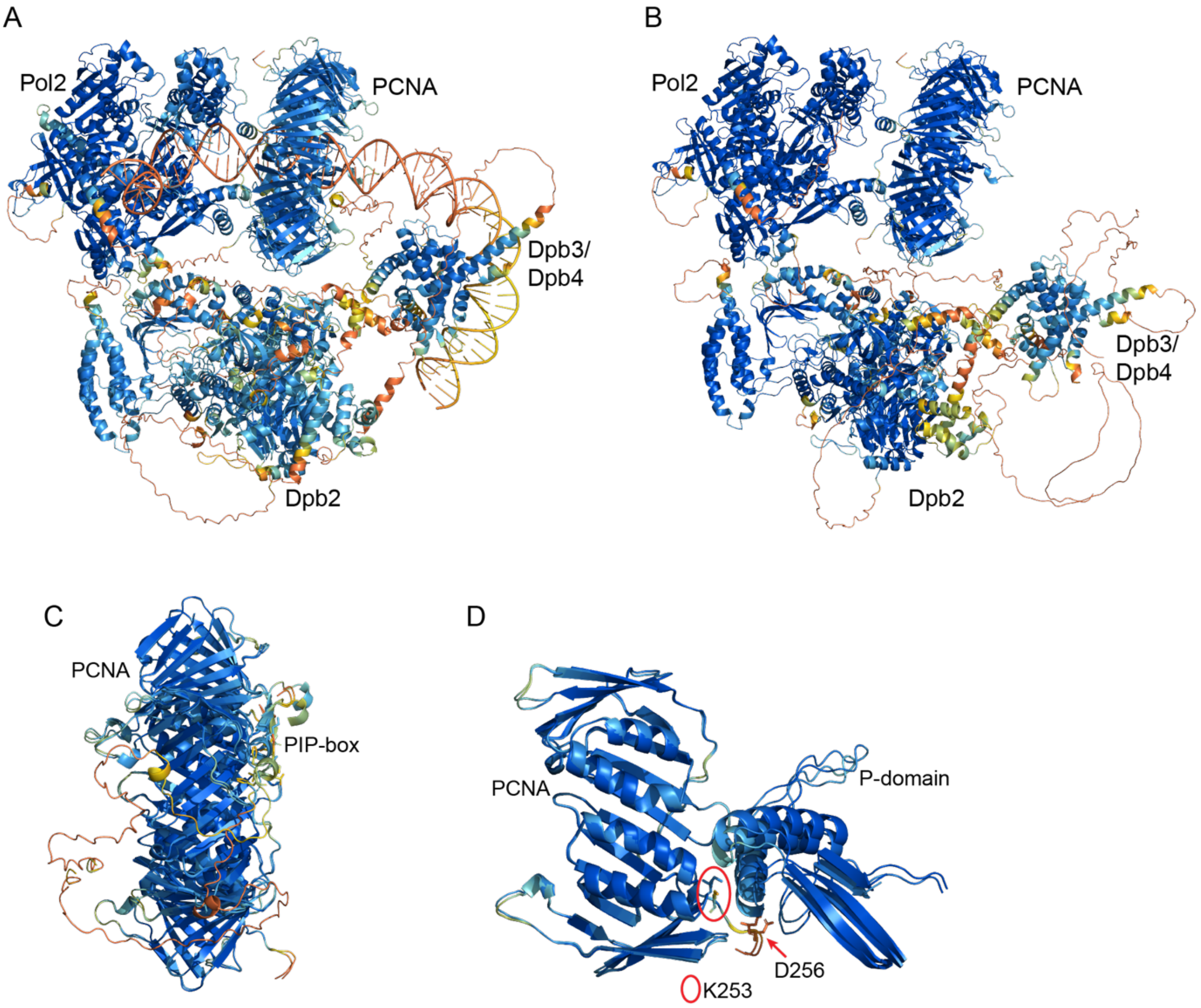

Supplementary Figure 2. Alphafold models colored according to the prediction confidence scores- dark blue (high), light blue (confident), yellow (low) and orange (very low). (A) Pol ε -PCNA-DNA complex as predicted by Alphafold3 and (B) Pol ε - PCNA complex as predicted by Alphafold2 C) zoomed in view of the PIP-box and linker region of the superimposed models of Pol ε -PCNA-DNA complex (Alphafold3) and Pol ε - PCNA complex (Alphafold2). D) zoomed in view of the interaction between P-domain and PCNA with superimposed models of Pol ε -PCNA-DNA complex (Alphafold3) and Pol ε - PCNA complex (Alphafold2).

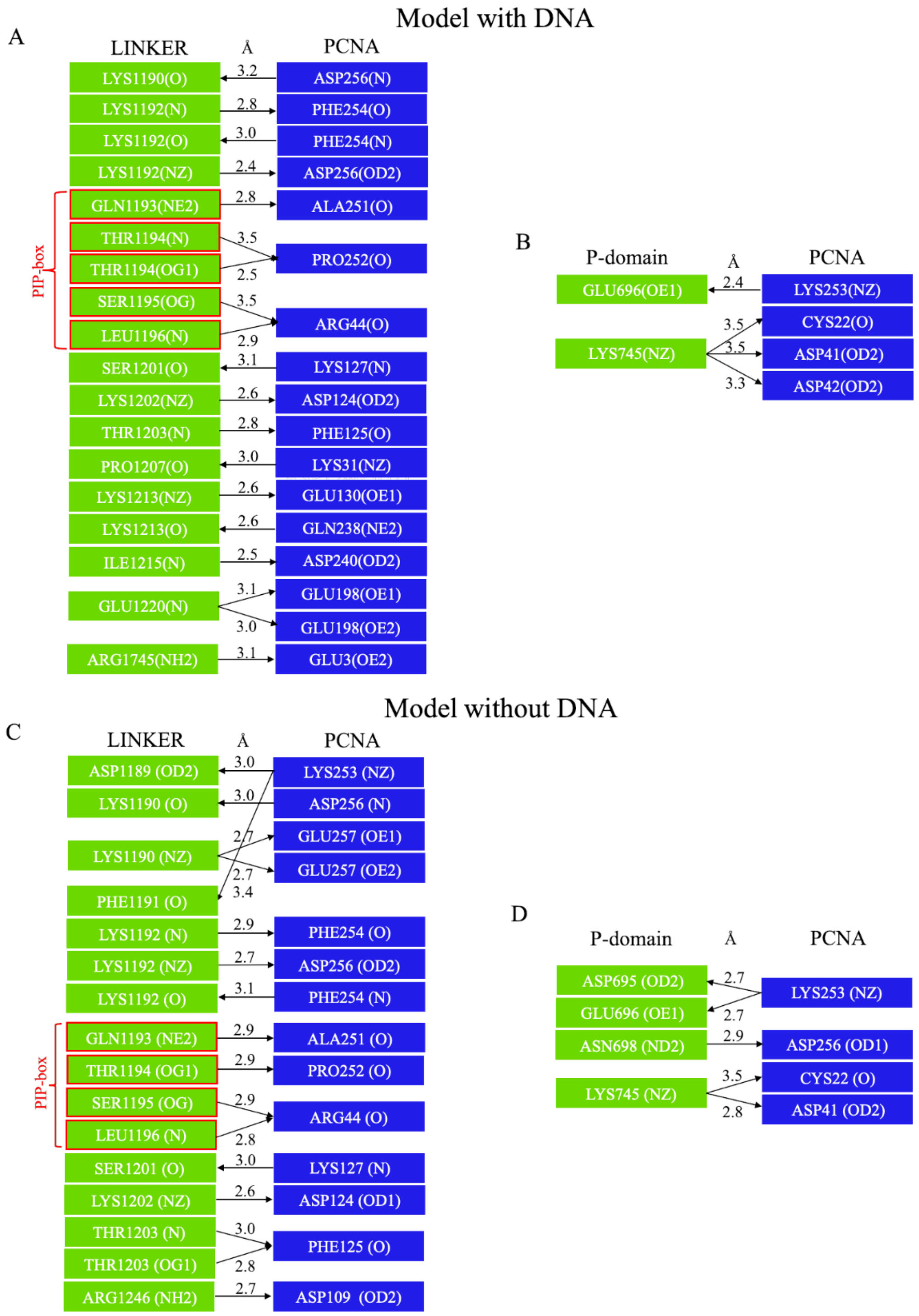

Supplementary Figure 3. Residues within H-bonding distance in (A and B) Pol ε -PCNA-DNA model as predicted by Alphafold3 and (C and D) Pol ε -PCNA model as predicted by Alphafold2. Green boxes denote residues in Pol ε and blue boxes denote residues in PCNA. The residues that constitute the classical PIP-box in Pol ε are shown with red outline.

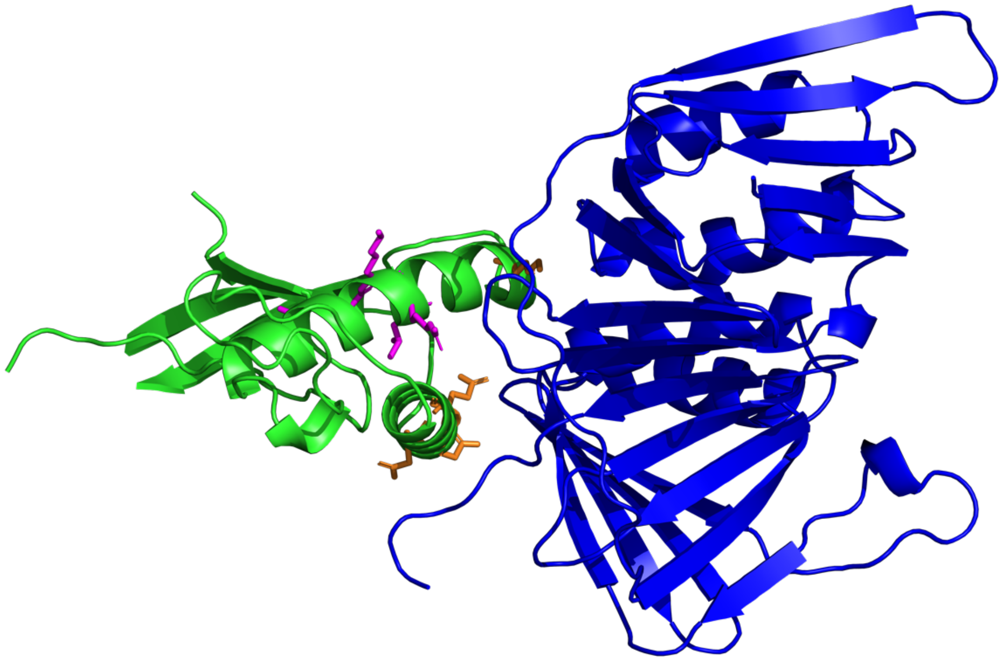

Supplementary Figure 4. Residues that are substituted in the P-domain. DENK-mutant ($D_{695}E_{696}N_{698}K_{745}$) shown in orange and P3 mutant ($K_{736}K_{737}R_{738}L_{739}$) shown in magenta. PCNA monomer is shown in blue.

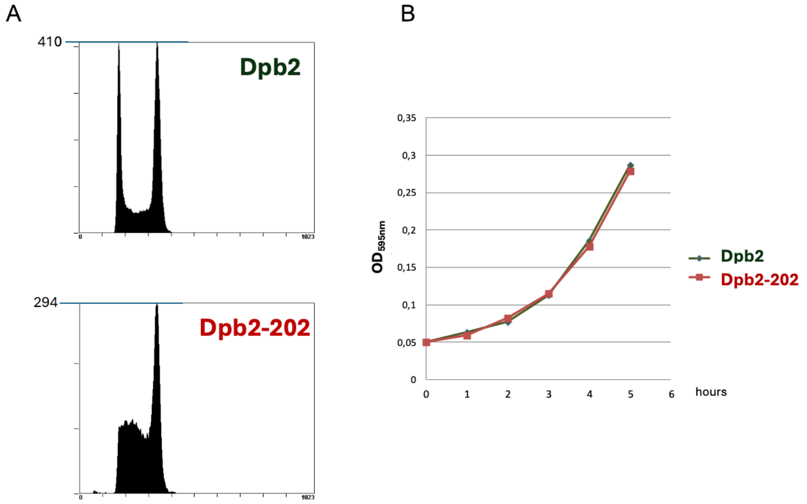

Supplementary Figure 5. (A)Flow cytometry analysis of an asynchronous haploid yeast culture with cells expressing either Dpb2 or dpb2-202.

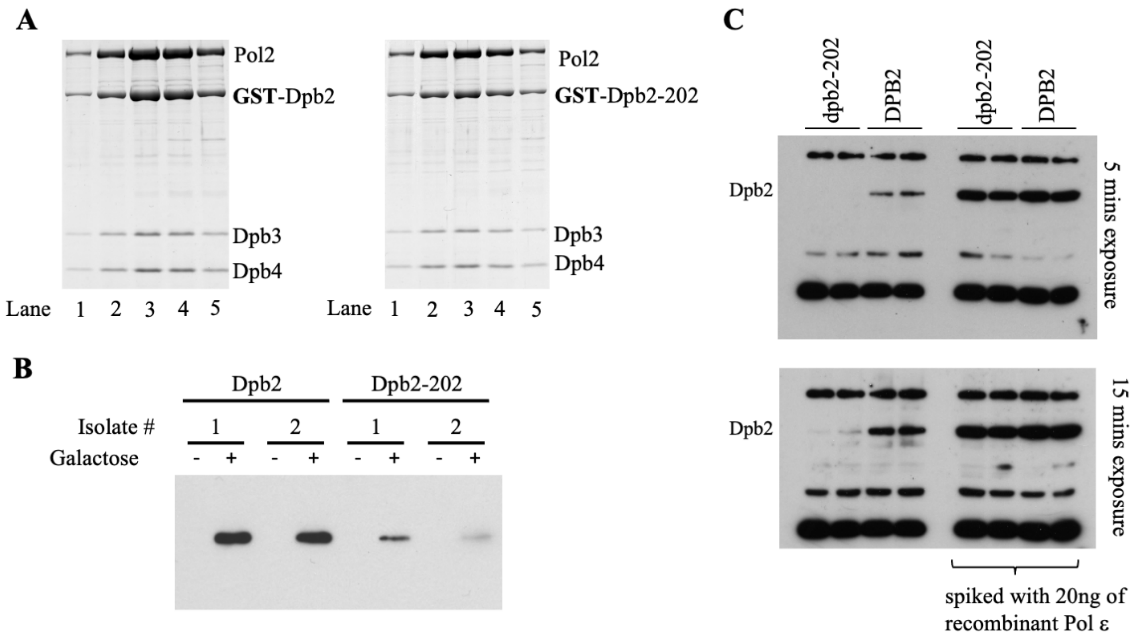

Supplementary Fig 6. (A) Eluate from Glutathione Sepharose with GST-Dpb2 or GST-Dpb2-202 in combination with recombinantly overexpressed Pol2, Dpb3 and Dpb4 in the yeast strain PY116. (B) Western blot of two PY116 cell isolates expressing GST-Pol2, Dpb2, Dpb3, Dpb4 and GST-Pol2, Dpb2-202, Dpb3, Dpb4 before and after induction with galactose showing presence of Dpb2 and Dpb2-202 in induced samples.(C)Western blot displaying *in vivo* levels of Dpb2 and Dpb2-202 in an asynchronous cell culture of the E134 strain, which carries either DPB2 or dpb2-202. Recombinantly expressed and purified Polε was added to the indicated lanes to illustrate the migration pattern of Dpb2 in the SDS-PAGE.

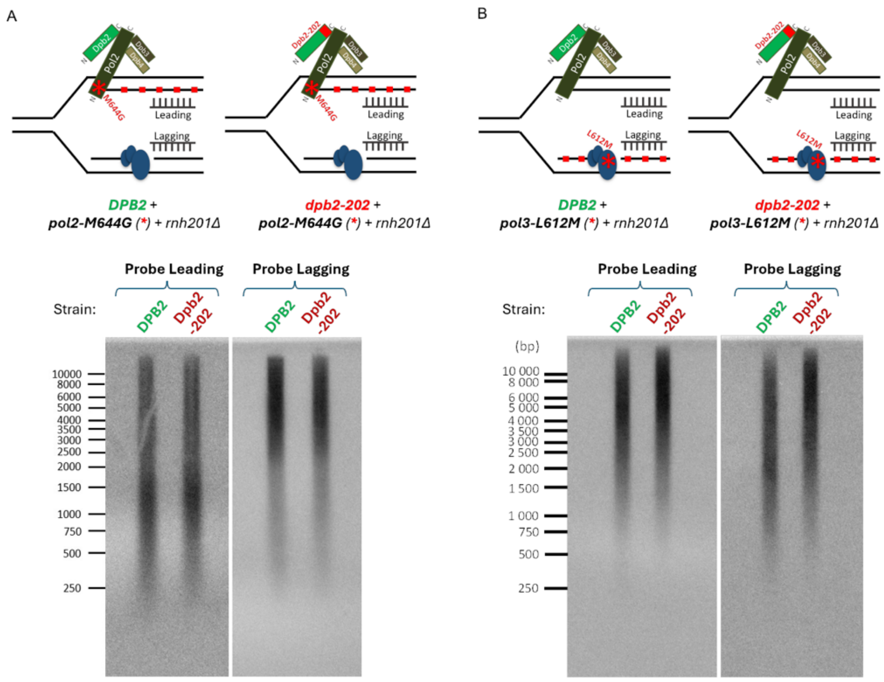

Supplementary Fig 7. Detection of strand specific incorporation of ribonucleotides using southern blot. (**A**) Detection of alkali-sensitive sites in *dpb2-202 pol2-M644G rnh201Δ* and *DPB2 pol2-M644G rnh201Δ strains*. (**B**) Detection of alkali-sensitive sites in *dpb2-202 pol3-L612M rnh201Δ* and *DPB2 pol3-L612G rnh201Δ* strains.

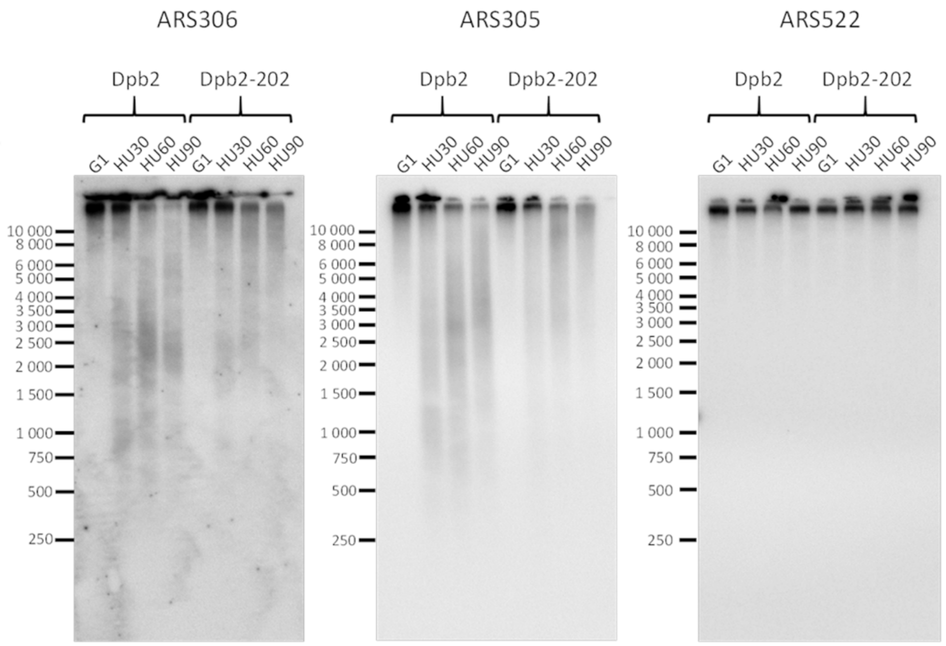

Supplementary Fig 8. Detection of replication intermediates using probes specific for ARS306, ARS305 and ARS522.

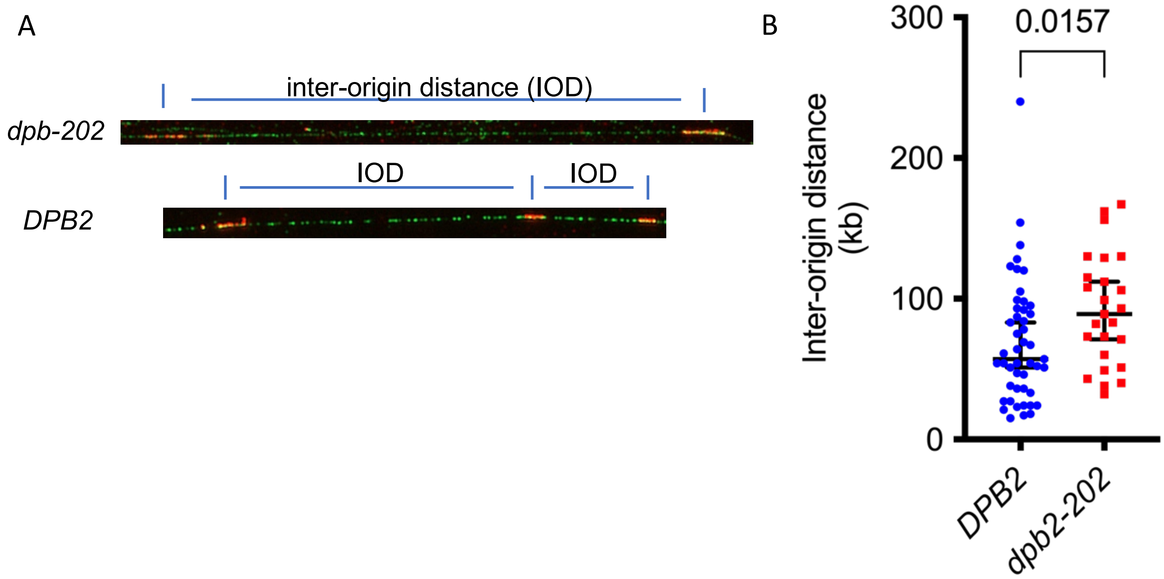

Supplementary Fig 9. Inter-origin distance in *DPB2* and *dpb2-202* cells inferred from DNA fiber analysis of BrdU incorporated DNA. A) representative images of inter-origin distances from *DPB2* and *dpb2-202* yeast strains. B) Measured inter-origin distances (kb) in the *DPB2* and *dpb2-202* yeast strains. Error-bars represent the median inter-origin distance with 95% confidence interval. The compared groups were found to be significantly different (P<0.05) based on an unpaired two-tailed Mann Whitney test (P=0.0157).

References

1. Chilkova, O., Jonsson, B.H. and Johansson, E. (2003) The quaternary structure of DNA polymerase epsilon from Saccharomyces cerevisiae. *The Journal of biological chemistry*, **278**, 14082-14086.
2. Sengupta, S., van Deursen, F., de Piccoli, G. and Labib, K. (2013) Dpb2 integrates the leading-strand DNA polymerase into the eukaryotic replisome. *Curr Biol*, **23**, 543-552.
3. Isoz, I., Persson, U., Volkov, K. and Johansson, E. (2012) The C-terminus of Dpb2 is required for interaction with Pol2 and for cell viability. *Nucleic acids research*, **40**, 11545-11553.
4. Santocanale, C. and Diffley, J.F. (1998) A Mec1- and Rad53-dependent checkpoint controls late-firing origins of DNA replication. *Nature*, **395**, 615-618.
5. Tanaka, S. and Araki, H. (2011) Multiple regulatory mechanisms to inhibit untimely initiation of DNA replication are important for stable genome maintenance. *PLoS Genet*, **7**, e1002136.
6. Ter Beek, J., Parkash, V., Bylund, G.O., Osterman, P., Sauer-Eriksson, A.E. and Johansson, E. (2019) Structural evidence for an essential Fe-S cluster in the catalytic core domain of DNA polymerase. *Nucleic Acids Res*, **47**, 5712-5722.
7. Burgers, P.M. (1999) Overexpression of multisubunit replication factors in yeast. *Methods*, **18**, 349-355.
8. Shcherbakova, P.V. and Kunkel, T.A. (1999) Mutator phenotypes conferred by MLH1 overexpression and by heterozygosity for mlh1 mutations. *Mol Cell Biol*, **19**, 3177-3183.

9. Aksenova, A., Volkov, K., Maceluch, J., Pursell, Z.F., Rogozin, I.B., Kunkel, T.A., Pavlov, Y.I. and Johansson, E. (2010) Mismatch repair-independent increase in spontaneous mutagenesis in yeast lacking non-essential subunits of DNA polymerase epsilon. *PLoS genetics*, **6**, e1001209.

10. Obi, I., Rentoft, M., Singh, V., Jamroskovic, J., Chand, K., Chorell, E., Westerlund, F. and Sabouri, N. (2020) Stabilization of G-quadruplex DNA structures in Schizosaccharomyces pombe causes single-strand DNA lesions and impedes DNA replication. *Nucleic Acids Res*, **48**, 10998-11015.
